# Innovative Approach Reveals How Receptors Control G Protein State Equilibria

**DOI:** 10.1002/advs.77969

**Published:** 2026-10-15

**Authors:** Alexandre Bouyssou, Mélanie Da Silva, Julien Soulé, Ludovic Fabre, Laurent Lamarque, Rémy Sounier, Thierry Durroux, Sébastien Granier, Thomas Roux, Elodie Dupuis, Eric Trinquet, Philippe Rondard, Jean‐Philippe Pin

**Affiliations:** ^1^ CNRS Institut De Génomique Fonctionnelle (IGF) Inserm Université De Montpellier Montpellier France; ^2^ Revvity Codolet France

**Keywords:** G protein empty state, G protein open state, GTP‐GDP exchange, opioid receptors

## Abstract

G protein‐coupled receptors (GPCRs) constitute the largest family of drug targets, acting through heterotrimeric G proteins. Agonist bound GPCRs promote GDP release from G proteins allowing their activation upon GTP binding. Here, we analyze this process with innovative tools that detect the main states of native Gi proteins: the GDP‐bound, the open nucleotide‐free and the active GTP‐bound states. We show that receptor activation increases the amount of open‐state Gi proteins, consistent with GDP release from inactive Gi proteins. We also observe that the active receptor can release GTP from the active Gi protein, to return to an inactive open state. Consistently, receptor activation also promotes the formation of the Gαβγ complex from the GTP‐bound state in cells, in agreement with the formation of the receptor‐G protein ternary complex. These data suggest that the amount of active GTP‐bound form produced upon GPCR activation can be controlled by the receptor‐induced GTP release. This control of G protein state equilibrium that depends on the receptor subtype may affect coupling efficacy. These data reveal receptor‐dependent G‐protein state equilibria and identify GTP release as an additional, context‐dependent control point that can shape apparent coupling efficacy.

## Graphical abstract

Using conformational specific trFRET biosensors of the Gi protein (depicted at the bottom), the authors show that receptor activation can not only release GDP from the inactive G protein, but can also release GTP from the activated G protein, leading to the reformation of the RG ternary complex. As such, under specific conditions, this property that depends on the receptor and G protein subtypes, may lead to a limit in the total amount of active G proteins produced by the receptor.

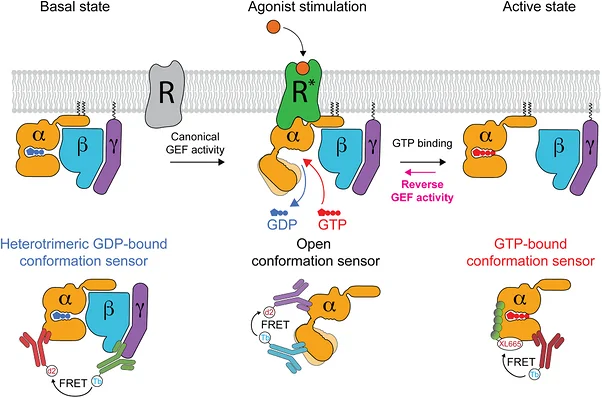

## 1 Introduction

G protein‐coupled receptors (GPCRs) are the largest family of cell surface receptors and are involved in many important physiological processes. For these reasons, GPCRs are the most important drug targets with approximately one‐third of all approved drugs acting on such receptors [1]. GPCRs activate several signaling cascades through various effectors including arrestins and heterotrimeric G proteins [2]. The molecular basis of G protein activation by GPCRs has been studied for many years [3, 4]. G proteins adopt three main conformational states, the inactive GDP‐bound, a nucleotide‐free open state [3, 4, 5, 6] and the active GTP‐bound conformations (Figure 1A). In the active state, GTP is rapidly hydrolyzed into GDP by the GTPase activity of the Gα subunit, a reaction catalyzed by RGS proteins [7]. The open, nucleotide‐free state of the G protein results from the formation of the high affinity ternary complex between the agonist, the receptor (R) and the Gαβγ complex after GDP release [8, 9]. While this R‐Gαβγ open complex can be stable in vitro [10, 11], it is transient in cells because of the intracellular concentration of GTP [12]. Structures of such ternary complexes have been solved for many receptors and G protein subtypes [8, 10], and revealed the open conformation of the Gα subunit. The empty open Gα subunit (Gα_OPEN_) has a high affinity for GTP, such that the complex R Gα_OPEN_βγ is destabilized by GTP binding, resulting in a large reorganization between Gα‐GTP and Gβγ.

**FIGURE 1:**
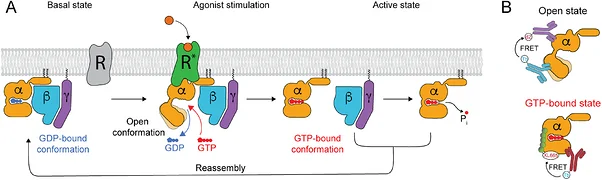
G protein activation cycle and FRET sensors developed. In the GPCR resting state, G protein is in GDP‐bound conformation composed by three subunits, Gα bound to GDP, Gβ and Gγ. Upon stimulation by an extracellular agonist, the receptor adopts a conformational change that allows G protein binding and catalyzes GDP release from Gα. Then the G protein is found in an empty conformation that leads to a high‐affinity complex between the activated receptor and the G protein. This high‐affinity complex is destabilized by GTP binding to Gα, that allows the Gα‐GTP and Gβγ subunits to interact with downstream effector proteins. Signaling from G protein is terminated on hydrolysis of GTP to GDP by Gα that is favored by the regulator of G protein signaling (RGS), then leading to a reassociation of the G protein heterotrimer. (B) G protein conformations detected by the FRET sensors developed in this study. Scheme adapted from Rasmussen et al., 2011 [8].

Importantly, while GPCRs are classically described as guanine nucleotide exchange factors that promote GDP release and subsequent GTP binding, early biochemical studies in rhodopsin/transducin, adrenergic and muscarinic systems indicated that receptor‐catalyzed nucleotide exchange can be reversible and that activated receptors can promote release of bound non‐hydrolysable GTP analogues [13, 14, 15, 16]. Subsequently this behavior was demonstrated in native cardiac and leukocyte membranes [17, 18]. This phenomenon, sometimes referred to as ‘reverse mode’ coupling, has recently regained attention through radioligand‐based pulse–chase studies at opioid receptors, which reported agonist‐dependent GTPγS release and proposed the existence of ligand‐selective receptor active states favoring GTP release vs. binding [19, 20].

GPCRs can activate multiple G protein subtypes (G_s_, G_i/o_, G_q_ and G_12/13_) with most receptors able to couple to several of these [21, 22, 23, 24]. The GPCR ability to activate multiple G protein subtypes and the possibility of developing drugs that favor the activation of only some of them, in a concept named biased signaling [25], underline the necessity of better understanding the way each G protein subtype is activated. Multiple assays measuring the activation of endogenous G proteins [10, 26, 27] that avoid the influence of overexpression of G protein sensors [28, 29, 30, 31, 32] are being used. They are based on the quantification of the active GTP‐bound G proteins through the measurement of GTPγS or fluorescent GTP binding [26, 27, 33, 34, 35]. However, these assays are not specific for one G protein subtype, unless an immunoprecipitation step is added [36]. Recently, the detection of the GTP‐bound form of Gα subunits through their interaction using specific sensors has been reported [30]. The sensor was modified to recognize and be specific to different G protein families. While these assays successfully track the final step in the activation process, they do not enable the analysis of the equilibrium between the three main conformations of the G protein upon receptor activation, an essential aspect to understand the effect of various ligands and receptors.

Here, we aimed at examining the equilibrium between the G protein states upon receptor activation. For this, we developed and characterized pairs of binders that selectively recognize either the open form, or the GTP bound form of native Gα_i_ (Figure 1B). After labeling with fluorophores compatible with time‐resolved Förster resonance energy transfer (trFRET) measurements, we could measure the appearance or disappearance of these different conformations of the endogenous Gα_i_ proteins upon receptor activation. Using these tools, we reveal that activated GPCRs can not only release GDP from the inactive state of Gα_i_ but also limit the amount of GTP‐bound form, by stabilizing the open state of the G protein. These findings reveal that the amount of active GTP‐bound form of the G protein can be regulated by the activated receptors, in both positive and negative ways. Such data suggest a more complex mode of G protein regulation by GPCRs and highlight the importance of considering the equilibrium between the different G protein states to understand the effects of various ligands and receptors on G protein signaling.

## 2 Results

### 2.1 Innovative Tools to Detect Gα_i_ Conformational States

Using the purified Gαi bound to GDP (Gαi1‐GDP) and the heterotrimer Gαi1‐GDP β_1_γ_2_, we have developed and characterized three kinds of binders: (1) a peptide that recognizes selectively Gi proteins in their active state, based on previously reported peptides [35]; (2) a commercial anti‐Gαi monoclonal antibody; (3) finally, home‐made monoclonal antibodies developed by mice immunization with the recombinant monomeric Gαi protein.

We have generated a 19‐residue peptide biotinylated at the N‐terminal and coupled to streptavidin labeled with the fluorophore acceptor XL665 compatible with trFRET measurement. This peptide, called KB1753, was reported to bind specifically the GTPγS bound conformation of the Gα_i_ protein family [37, 38]. To improve its apparent affinity, we have prepared a biotinylated version that can be detected with a multivalent and fluorescent streptavidin. This peptide, called KB1753DS, and referred as peptide‐XL665, binds selectively to the active conformation of Gα_i1_ proteins stabilized by GTPγS, but not to its GDP and empty conformations (Figure 2A). The trFRET signal measured, in a homogenous configuration (HTRF), between increased amounts of the XL665‐labelled peptide in presence of a constant concentration of the commercial monoclonal antibody Ab13 labelled with the fluorescent donor Lumi4‐Tb. Of note, Ab13 has a similar affinity for the different conformations of Gα_i1_, GDP, GTPγS and empty state, as measured by the FRET signal between d2‐labelled Ab13 and the anti‐Twin‐Strep‐tag (TST) antibody labeled with Lumi4‐Tb that recognizes a TST‐tag introduced at the N‐terminal part of the Gα subunit (Figure 2B).

**FIGURE 2:**
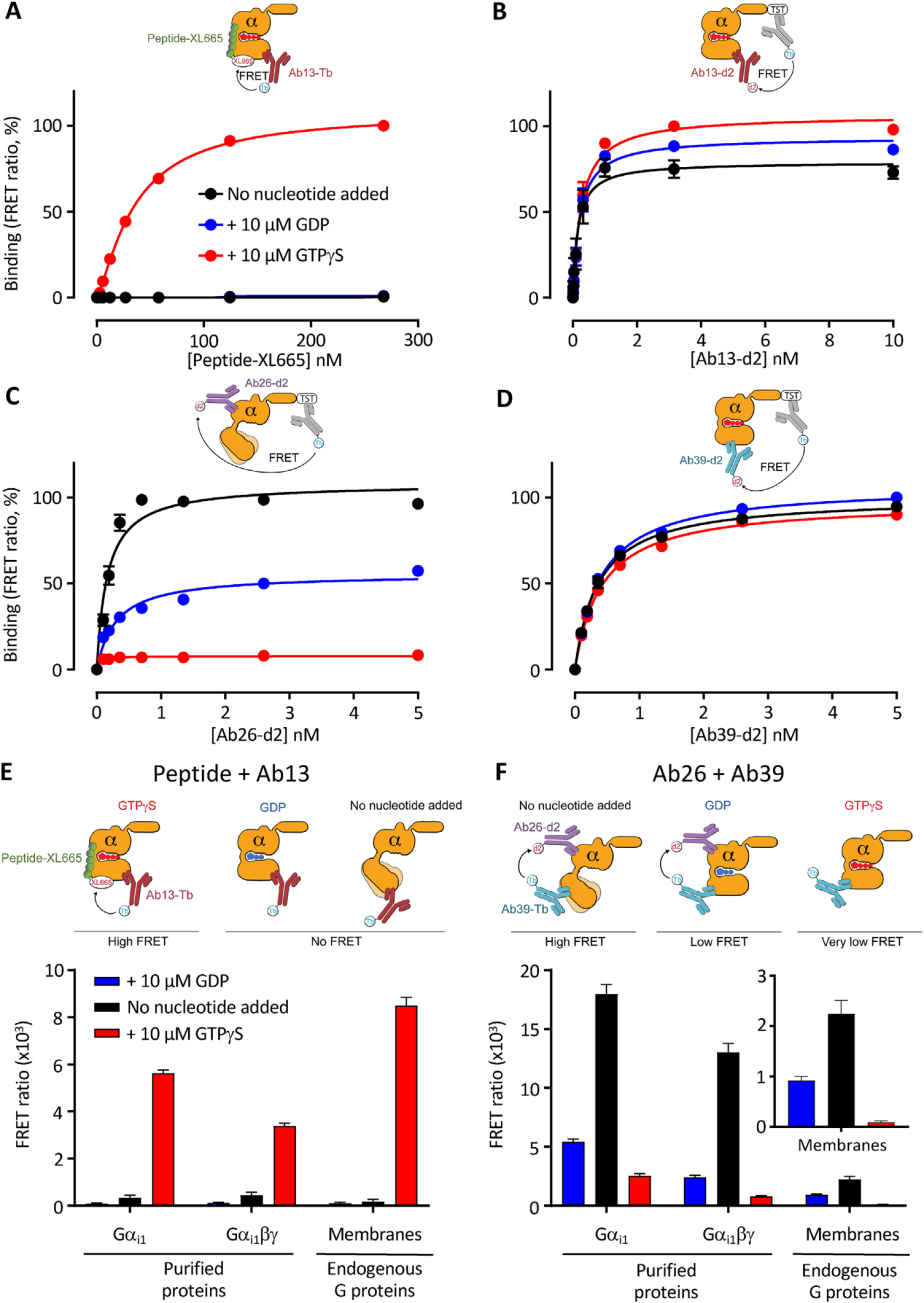
Tools for detecting Gα_i1_ subunit and development of FRET‐based conformational sensors. (A) The peptide KB1753DS labeled with the acceptor XL665 is specific of the active conformation, or GTPγS‐bound conformation, of Gα_i1_. Binding of the peptide on the purified TST‐Gα_i1_ protein incubated with or without the indicated nucleotides (10 µM) is measured by trFRET using the antibody Ab13 labeled with a donor. (B) Binding of the antibody Ab13 labelled with the acceptor (d2) on the purified TST‐tagged Gα_i1_ protein bound to an anti‐TST antibody labeled with a donor (Tb) in absence or presence of the indicated nucleotides (10 µM), and measured by trFRET. (C,D) Binding of the antibody Ab26 *(C)* and Ab29 *(D)* in similar conditions than in *panel B*. (E) FRET sensor developed using the pair composed by the peptide labeled with acceptor and the Ab13 labeled with donor. It is specific of the active conformation, GTPγS‐bound conformation, of the purified G proteins, either Gα_i1_ alone or the heterotrimer Gα_i1_βγ, and of the endogenous G proteins found in cellular membrane preparations. Values are means ± s.e.m. of three independent experiments performed in triplicates. (F) FRET sensor made of the antibodies Ab26 labeled with acceptor and Ab39 labeled with a donor. It is specific of the open state, or nucleotide free form, with a low trFRET signal for the inactive state, GDP‐bound conformation, and almost no trFRET signal for the active state, GTPγS‐bound conformation. Values are means ± s.e.m. of three independent experiments performed in triplicates.

We have also developed additional monoclonal antibodies, by immunizing mice with purified recombinant Gα_i1_ loaded with either GDP or GTPγS, because the nucleotide‐free or open state of Gα (Gα_OPEN_) was proposed to be thermally unstable [37]. Among them, Ab26 is selective of both the GDP and Gα_OPEN_ conformations, and it did not bind to the GTP state (Figure 2C). The higher maximum FRET signal for Gα_OPEN_ than Gα_i1_‐GDP was most probably due to a larger distance between the two antibodies rather than from a difference of affinity. Indeed, Ab26 has a similar Kd value for Gα_OPEN_ and Gα_i1_‐GDP (Table S1). In contrast, Ab39 has no preference for the different Gαi protein conformations (Figure 2D), similarly to the Ab13 described above. Of note, the peptide has a nanomolar affinity for Gα_i1_‐GTPγS while these monoclonal antibodies have a sub‐nanomolar affinity for the different conformations of Gα_i1_ (Table S1).

### 2.2 FRET Sensors to Detect Conformational States of Endogenous Gi Proteins

We combined these binders in order to develop trFRET conformational sensors to detect the endogenous Gα_i1_‐GTPγS and Gα_OPEN_ states of the Gα_i_ proteins. The pair peptide‐XL665 + Ab13 was specific of the GTPγS active, for both the purified Gα_i1_ and the heterotrimer Gα_i1_. β_1_γ_2_ (Figure 2E). It suggested that GTPγS was able to dissociate the Gβγ subunits from Gα thus removing its steric hindrance for the binding of the peptide known to bind to the Switch 2 region of Gα [37]. Most importantly, the peptide‐XL665 and Ab13 allowed the detection of endogenous Gi proteins in cell membrane preparations with high sensitivity (Figure 2E).

Detecting the Gα_OPEN_ could be a highly efficient approach to reveal the activation of G protein by the receptor [39]. Interestingly, the pair Ab26 + Ab39 recognized most preferentially the Gα_i_‐_OPEN_ states both for the purified Gα_i_ and Gα_i_βγ and the endogenous Gi proteins in membranes (Figure 2F). Of note, the low level of FRET signal obtained with the membranes might be due to a lower amount of Gi proteins in the assays compared to the recombinant Gi proteins, or alternatively to steric hindrance limiting the binding of at least one antibody, coming from the receptor or other components of the membranes. Nevertheless, the lower endogenous level of Gi proteins did not prevent the specific detection of Gα_OPEN_ (Figure 2F, inset), further indicating the high sensitivity of this FRET sensor. Finally, the detection of Gα_i_‐_OPEN_ by this pair of antibodies was further confirmed by treatment with apyrase, an enzyme that hydrolyzes GDP into GMP which has a low affinity for the G protein [8, 9, 40]. Apyrase strongly increased the FRET signal of the pair Ab26 + Ab39 for both the recombinant Gα_i_ and endogenous G proteins loaded with GDP, to a level similar to that obtained in the absence of added nucleotide (**Extended Data** Figure 1). In addition, a similar FRET signal for the monomeric recombinant Gα_i_ in the absence or presence of apyrase indicated that this protein purified in absence of nucleotide was most probably in the nucleotide free open state.

### 2.3 Specific Gα_i_ Subunits are Detected by the Biosensors

We have determined which G protein subtypes are detected by our conformational sensors. To do so, we have developed a cellular assay where the binding of the antibodies was measured for each transfected Gα subunit tagged with both a TST and a Flag epitope at their N‐terminus (**Extended Data** Figure 2A). After HEK293 cells permeabilization, trFRET signal was measured between the anti‐TST antibody labeled with Lumi4‐Tb, and the antibodies Ab13, Ab26 or Ab39 labeled with d2 for each transfected Gα subunit (**Extended Data** Figure 2B–D). As a control, we verified that all the Gα subunits were expressed at similar level, except Gα_12_ expressed at a lower level (**Extended Data** Figure 2E,F), by measuring the FRET signal between the anti‐TST‐Lumi4‐Tb and anti‐Flag‐d2 antibodies. A similar trFRET‐based method for the relative quantification of proteins, including G proteins and GPCRs, in biological samples has been reported [41, 42].

Interestingly, Ab13 is highly selective for Gα_i1_ and Gα_i3_ and Ab39 of Gα_i1_ in these cellular assays (**Extended Data** Figure 2B,C). Ab26 recognized preferentially both Gα_i1_ and Gα_i3_ subunits, but a low FRET signal was also observed with other G_i/o_ subunits such Gα_i2_, Gα_o_ and Gα_z_ (**Extended Data** Figure 2D), suggesting it binds these subunits with a lower affinity than Gα_i1_ and Gα_i3_. Of note, the binding of Ab13 and Ab39 was not sensitive to the conformation of the Gα subunits, since the addition of saturating concentrations of GDP or GTPγS did not modify the FRET signal (**Extended Data** Figure 2B,C). In contrast, Ab26 better recognized the “no nucleotide added” and GDP‐bound states than the GTPγS bound state (**Extended Data** Figure 2D), as shown above with the purified G protein (Figure 2C). Finally, the peptide KB7153DS was previously reported to be specific of the GTPγS active state of Gα_i1_, Gα_i2_ and Gα_i3_, but it did not recognize the Gα_o_ subunit [43].

Altogether, we conclude that the FRET sensor made of Ab13 + peptide can detect specifically the GTP bound state of Gα_i1_ and Gα_i3_ in membranes (Figure 2E). The pair Ab26 + Ab39 is specific of the endogenous Gα_i1_, indicating this latter is well expressed in the HEK293 cell membranes used (Figure 2F). Of note, the stronger FRET signal of Ab13 + peptide‐XL665 compared to Ab26 + Ab39 in membranes (Figure 2E,F), might be due to the presence of Gα_i3_ subunits in the membrane fractions [43, 44]. But alternatively, this better detection of the endogenous Gα_i1_ by the pair Ab13 + peptide might be due to a lower steric hindrance of the tools with the membranes compared to the pair of antibodies. Although we cannot exclude a higher efficacy of FRET for the pair Ab13 + peptide when bound to the Gα protein.

### 2.4 Sensors Detect the Relative Reorientation of the α‐Helical and Ras‐Domains

In order to better understand the molecular basis of these sensors, we have performed an epitope mapping of the three antibodies based on their high selectivity for some Gi proteins. First, overlapping epitopes between Ab13 and Ab39 have been proposed based on competition assays (**Extended Data** Figure 3). Briefly, the FRET binding saturation experiments of the antibodies Ab13, Ab26 and Ab39 have been performed in presence of one of the unlabeled G protein binders (Ab13, Ab26, Ab39 or the peptide‐XL665). A strong competition was measured between Ab13 and Ab39, and not between the other binders, and it was consistent with a direct competition.

**FIGURE 3:**
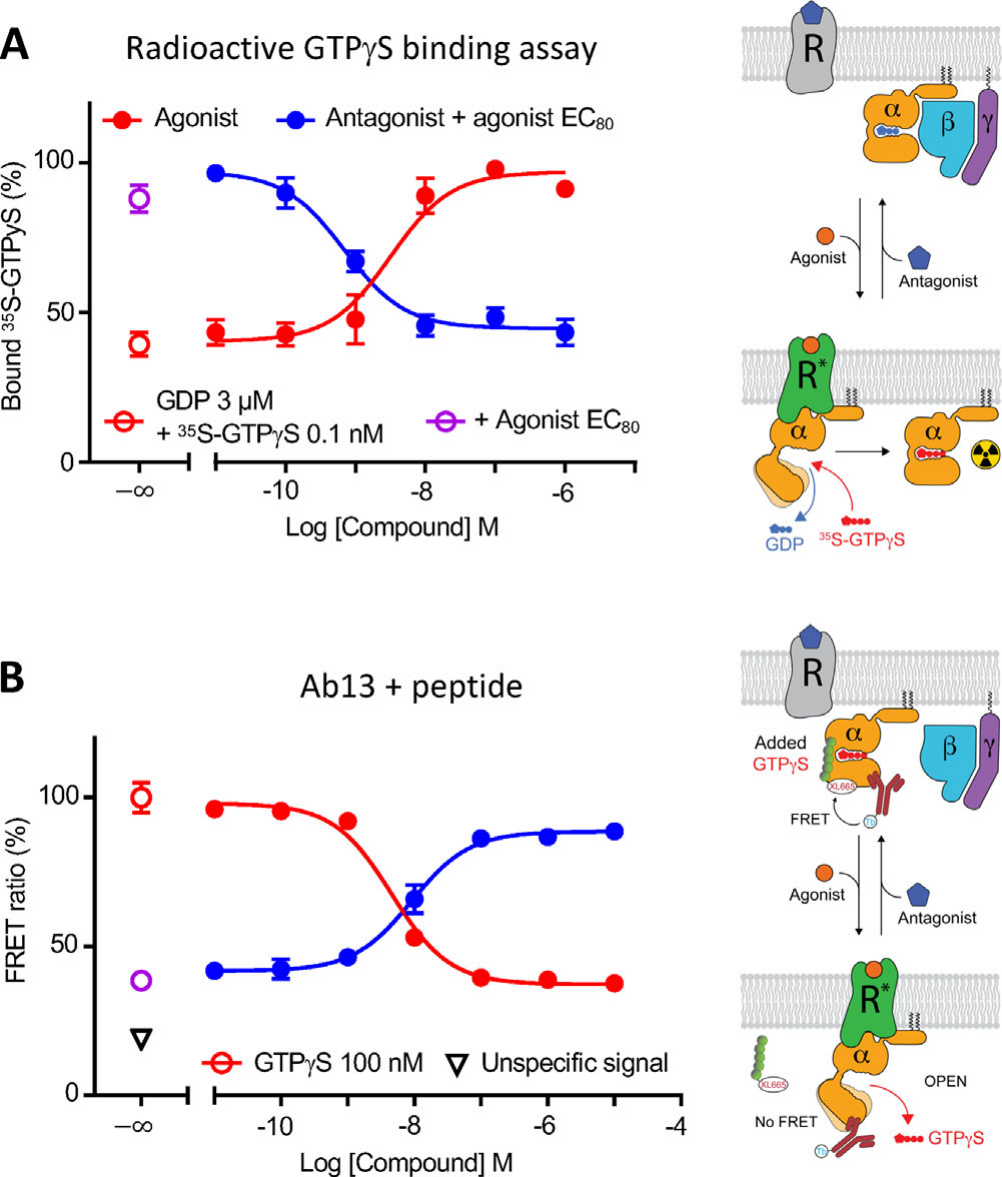
The FRET sensor measures the activation of G_i_ proteins by the activated GPCR through the accumulation of G_i_ open state. (A) Measurement of the activation of the G proteins by the human δ‐opioid receptor in presence of the agonist SNC162 (pEC50 = 8.52 ± 0.09), or the antagonist naltrindole (pIC50 = 9.15 ± 0.07), in membrane preparations with the classical [35S]‐GTPγS binding assay. Values are means ± s.e.m. of three independent experiments performed in triplicates. A scheme illustrating the binding of [^35^S]‐GTPγS is shown. (B) Measurement of the activation of the endogenous Gi proteins by the human δ‐opioid receptor in presence of the agonist SNC162 (pEC50 = 8.09 ± 0.02), or the antagonist naltrindole (pIC50 = 7.91 ± 0.02), in membrane preparations, using the FRET‐based Gi protein sensor made of the peptide‐XL665 and the Ab13 labeled with a donor. The experiments were performed in presence of 100 nM GTPγS and 10 ng of membrane preparations. Values are means ± s.e.m. of three independent experiments performed in triplicates. A scheme illustrating the release of GTPγS is shown.

We then used bioinformatics analysis using the Gα_i_ structure and Gα protein sequence alignment to build six different Gα_i1_ mutants made of single or multiple changes (**Extended Data** Figure 4). Among them, one mutant (V109A, G112C and T120P) in the α‐helical domain abolished Ab39 binding and decreased the affinity 20‐fold of Ab13, while Ab26 binds with a similar affinity to both the mutant and wild‐type Gα_i1_ subunit. It is in agreement with Ab39 and Ab13 being competitive for the binding to Gα_i1_ by sharing overlapping epitopes, and also to their absence of sensitivity to the nucleotide bound. Regarding Ab26, one mutation (A59N) located in the linker connecting the GTPase domain and the α‐helical domain, impairs its affinity by a factor of 20. It is consistent with Ab26 being sensitive to the nucleotide bound, with a higher affinity for the GDP and empty Gα_i1_. Altogether, our results enable to propose a rough model for the epitope of the three antibodies. Ab13 and Ab39 would bind to α‐helical domain then compatible with the antibodies being far apart from the membrane, whereas Ab26 would bind between the two structural domains of Gα_i1_. These results of epitope mapping indicate that our two FRET sensors detect the relative reorientation between the α‐helical and ras‐domains that is expected to occur during the nucleotide exchange, and consistent with the appearance of a nucleotide‐free open conformation [4].

**FIGURE 4:**
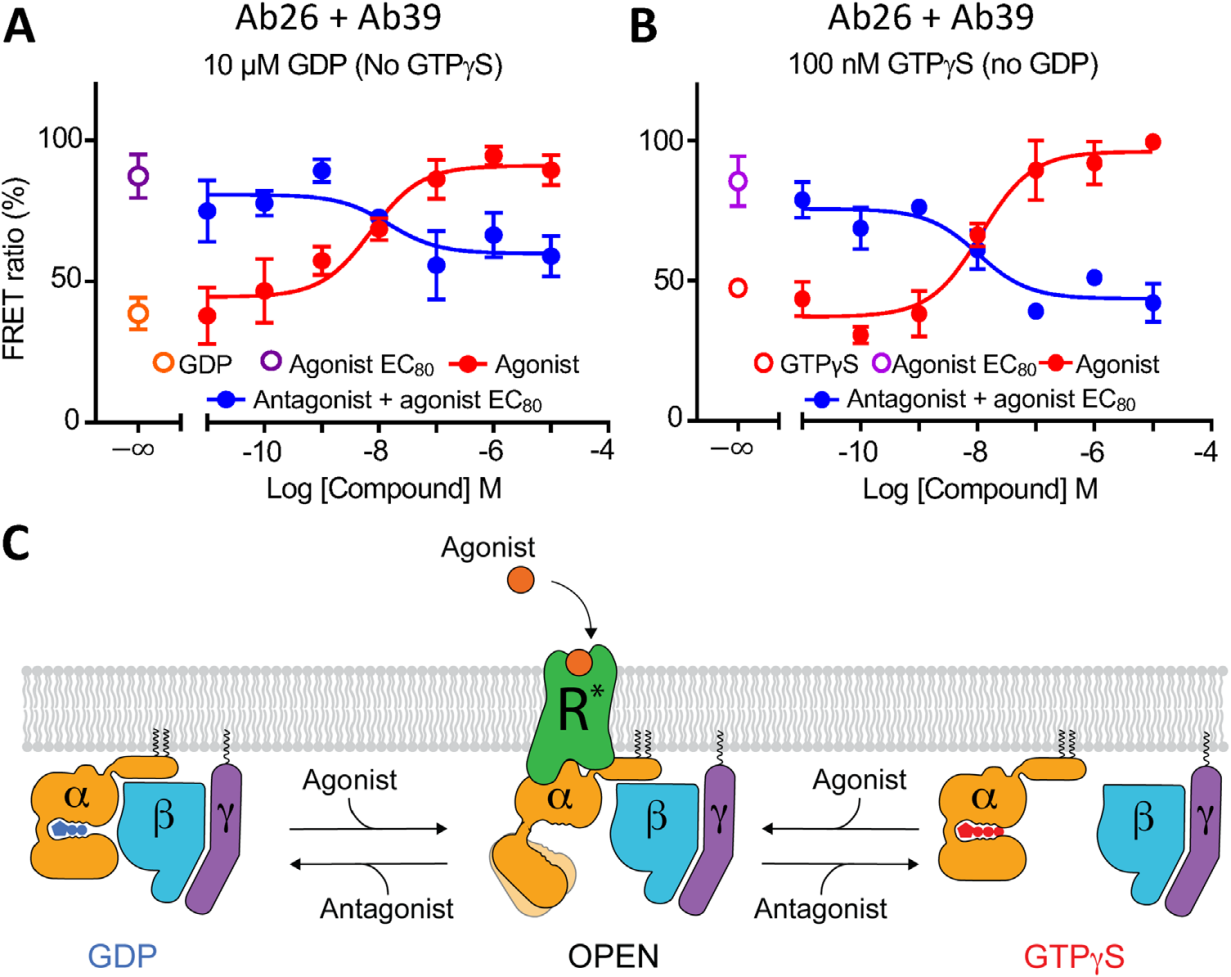
A FRET sensor specific of the open state of the Gi protein measures its accumulation by the activated GPCR. (A,B) Activation of the endogenous G_i_ proteins by the human δ‐opioid receptor in presence of the agonist SNC 162, or the antagonist naltrindole, using the FRET‐based G_i_ protein sensor made of the antibody Ab26 labeled with acceptor and the Ab39 labeled with donor. The experiment is performed in presence of 10 µM GDP *(A)* or 100 nM GTPγS *(B)* using 10 ng of membrane preparations. Values are means ± s.e.m. of three independent experiments performed in triplicates. (C) Schematic representation of the model proposed for G protein activation, where a stimulated GPCR is able to stabilize the endogenous G protein in the open state, either from the GDP‐bound or GTPγS‐bound conformation.

### 2.5 Monitoring Agonist‐Induced Gi Open State

We used these two FRET sensors to measure the activation of endogenous Gi proteins by a GPCR in membrane preparations. The human δ opioid receptor (DOP) known to couple to G_i/o_ proteins was used as a model. We first performed G protein activation with the sensor Ab13 + peptide at high concentration of nucleotides (10 µM GDP in the presence of 100 nM GTPγS) similar to the conditions used for GTPγ^35^S binding (Figure 3A). Surprisingly, the agonist SNC162 induced a strong decrease of FRET signal, while the antagonist naltrindole, in presence of EC_80_ of agonist, had the opposite effect (Figure 3B). Although SNC162 and naltrindole produced the expected agonist and antagonist profiles in both formats, their relative apparent potency values slightly differ between the GTPγ^35^S and the FRET assays, reflecting assay‐specific operational readouts rather than changes in intrinsic ligand affinity. The GTPγ^35^S assay measures bulk nucleotide incorporation under a high GDP:GTPγ^35^S ratio, which favors GDP‐loaded heterotrimers and may limit the contribution of receptor‐associated nucleotide‐free intermediates. In contrast, the GDP‐free FRET assay selectively monitors loss of Gα_i_‐GTPγS state and may allow a greater contribution from pre‐associated or nucleotide‐free receptor‐G protein complexes, thereby favoring agonist occupancy and reducing the apparent potency of antagonist displacement. Differences in assay sensitivity, incubation conditions and state kinetics, together with possible effects of the Ab13 + peptide pair on GTPγS association or dissociation, may also contribute to the observed shifts. Since Ab13 + peptide is specific to the Gα_i_‐GTPγS conformation, it suggests the agonist induced the accumulation of a Gα_i_ state different from the GTPγS conformation, most probably the Gα_OPEN_ conformation. Altogether, these data suggested that the agonist stabilized the nucleotide free state of the G protein, while the antagonist has an opposite effect by limiting the appearance of this open state (Figure 3B).

To further demonstrate this mechanism, we used the sensor Ab26 + Ab39 that detects specifically Gα_i‐OPEN_. Interestingly, a strong increase of FRET signal was measured in the presence of agonist and GDP but without GTPγS (Figure 4A). A similar effect of the agonist was obtained under conditions where GTPγS is present but not GDP (Figure 4B). It revealed that upon agonist stimulation, the receptor is able to stabilize the open state of the G protein, whether it was bound to GDP or GTPγS. As expected, the process was reversible since the antagonist, tested at EC_80_ agonist, reverses the high FRET signal (Figure 4A,B). It showed then that stabilizing the resting state of the receptor limits the accumulation of the G protein open state, in agreement with the ternary complex model where the agonist stabilized the receptor coupled to the nucleotide free G protein [9]. In the GDP condition, antagonist treatment did not fully return the trFRET signal to the baseline. This likely reflects both the narrower dynamic range of the Ab26 and Ab39 sensor with GDP because GDP‐bound Gαi retains measurable signal, whereas GTPγS‐bound Gαi is nearly undetected and the distinct equilibria imposed by the two nucleotides. GDP supports continued cycling between GDP‐bound and nucleotide‐free receptor‐coupled states, whereas GTPγS stabilizes a low‐signal GTP‐like state; thus, the residual signal cannot be assigned unambiguously to a single G‐protein population.

Altogether, these data demonstrated that agonists, by activating the receptor DOR, were able to favor the Gα_i‐OPEN_ state whatever the G protein was loaded with GDP or GTPγS. The most possible mechanism is that the receptor was able to empty the Gα subunit by inducing nucleotide release whatever the guanine nucleotide bound (Figure 4C). Conversely, and as expected for a reversible process, the antagonist produced the opposite effect by reducing the population of G protein existing in the empty state even in the presence of saturating concentrations of GDP or GTPγS or both.

### 2.6 Relative Quantification of the Agonist‐Induced Gi Open State

We have quantified the proportion of endogenous G proteins activated by the DOR agonist in membranes by measuring the accumulation of Gα_OPEN_. Using the sensor Ab13 + peptide‐XL665, for various concentrations of GTPγS (in the absence of GDP), we have measured the fraction of Gα_i_‐GTPγS either after spontaneous incorporation of GTPγS, or after agonist‐induced activation that stimulates GTPγS release (Figure 5). The difference between these two conditions enables the determination of the fraction of Gα_OPEN_ state induced by the agonist for each concentration of GTPγS.

**FIGURE 5:**
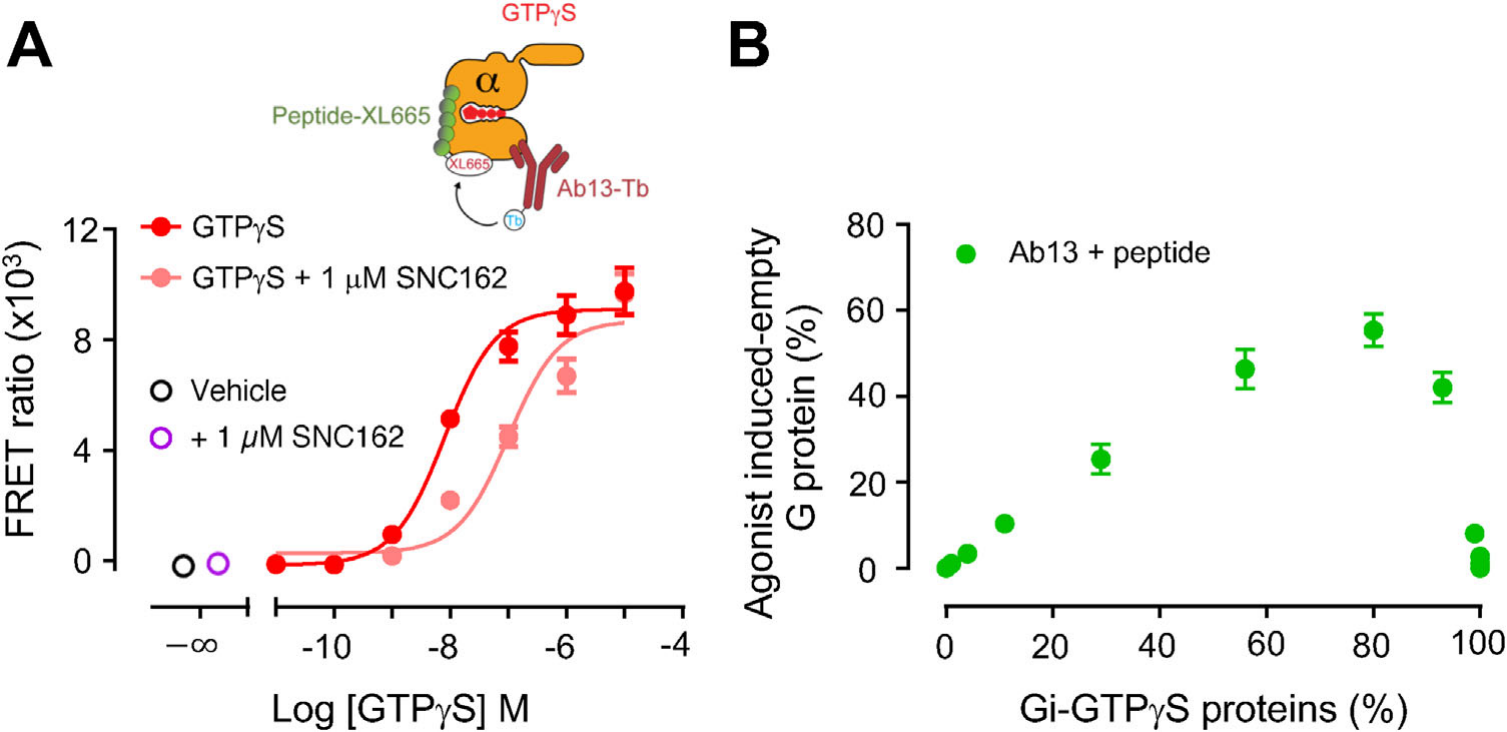
Determination of the amount of agonist‐induced Gα_i_ empty forms using the FRET sensors. (A) Detection of the endogenous Gα_i_ in a GTPγS‐bound conformation in the human δ‐opioid receptor expressing membrane preparations by the indicated FRET sensor for different amounts of GTPγS, in the absence or presence of the agonist SNC 162. (B) Rate of the agonist‐induced Gα_i_ empty determined from data in *panel A* as a function of the total amount of Gα_i_ in a GTPγS‐bound conformation also determined in *panel A* by the FRET signal between Ab13 and the peptide KB1753. Values are means ± s.e.m. of three independent experiments performed in triplicates.

In these experiments, the proportion Gα_OPEN_ state was determined by calculating the nucleotide site occupancy as a function of the affinity of GTPγS for the G protein as described below. We can then determine a factor that corresponds to the percentage of Gα_OPEN_ relative to the amount of total G (𝐺𝑡).

$$Kd=\frac{\left[L\right]\left[R\right]}{\left[RL\right]}$$

Given 𝑅𝑡 = 𝑅 + 𝑅𝐿

$$\left[RL\right]=Rt\left(\frac{1}{1+\frac{Kd}{\left[L\right]}}\right)$$

$$\left[RL\right]=Rt\frac{\left[L\right]}{\left[L\right]+Kd}$$

Given 𝐿 + 𝑅 = 𝑅𝐿, therefore GTPγS (= GTP) + 𝐺 = 𝐺_GTP_

𝐾𝑑 = GTP affinity for 𝐺, and 𝐿 = [GTP], then 𝐿 = 𝑥 𝐾𝑑

$$\left(G_{GTP}\right)=Gt\frac{x\;Kd}{\left(x+1\right)Kd}$$

$$\left(G_{GTP}\right)=Gt\frac{x}{\left(x+1\right)}$$

𝑥 / (𝑥 +1) = factor or rate of 𝐺*_GTP_* (fraction of Gα_i_.GTPγS for each concentration of GTPγS) relative to the total amount of G proteins (𝐺𝑡).

The rate of 𝐺*_GTP_* was then determined by measuring the Gα_i_‐GTPγS form using the sensor Ab13 + peptide, for each concentration of GTPγS, both for the spontaneous incorporation and the agonist‐induced conditions (Figure 5A). Interestingly, the proportion of agonist‐induced Gα_OPEN_ increased with the concentration of the Gα_i_‐GTPγS, until reaching a maximum of around 60% of the pool of endogenous and detectable Gi/o proteins (Figure 5B). Indeed, the assay resolves receptor‐driven redistribution between GTPγS‐bound and nucleotide‐free states over a dynamic range of approximately 1 nM to 1 µM GTPγS. Above this range, increasing nucleotide occupancy shifts the equilibrium in favor of GTPγS‐bound conformation, reducing our tools capacity to allow the detection of the agonist‐induced empty state (Figure 5A).

### 2.7 Pharmacological Characterization of Different G Protein‐Coupled Receptors

We then took advantage of our membrane‐based assay that relies on the detection of the endogenous Gα_OPEN_, to analyze the molecular pharmacology of different Gi‐coupled GPCRs [22, 32, 45]. These receptors are in different cellular backgrounds that can potentially have different composition of Gi protein subtypes [22, 46, 47]. We thus verified that the sensor Ab13 + peptide‐XL665 produced similar results in membrane preparations of the recombinant human DOR from HEK293, Chinese hamster CHO‐K1, and rat CHEM‐1 cell lines (**Extended Data** Figure 5). Similar trFRET signal changes for the agonist (SNC162) and antagonist (naltrindole) were obtained indicating our tools are not selective of the human G_i/o_ proteins but can recognize other mammalian species.

We then used our sensor Ab13 + peptide to perform the pharmacological characterization of DOR agonists (Figure 6A) and antagonists (Figure 6B) with different efficacies. The results confirmed that we can precisely define the potency of either agonists or antagonists of a given Gi‐coupled receptor. Indeed, pEC_50_ and pIC_50_ obtained for these compounds with our FRET sensor strongly correlated with those reported previously by [^35^S]‐GTPγS binding assay (Figure 6C,D). Of note, while the pIC_50_ for the antagonists were the same in the two assays (Figure 6D), a shift to better potencies was measured with the agonists in our FRET sensor assay (Figure 6C). It might be due to the influence of the tools used to detect the Gi.GTPγS conformation, since Ab13 increased the kinetics of GTPγS‐Bodipy binding (k_on_) and the releasing kinetic (k_off_) of GTPγS by the Gα_i_ subunit, alone or in presence of the peptide‐XL665 (**Extended Data** Figure 6A), while this effect was less observed with the other antibodies (**Extended Data** Figure 6B).

**FIGURE 6:**
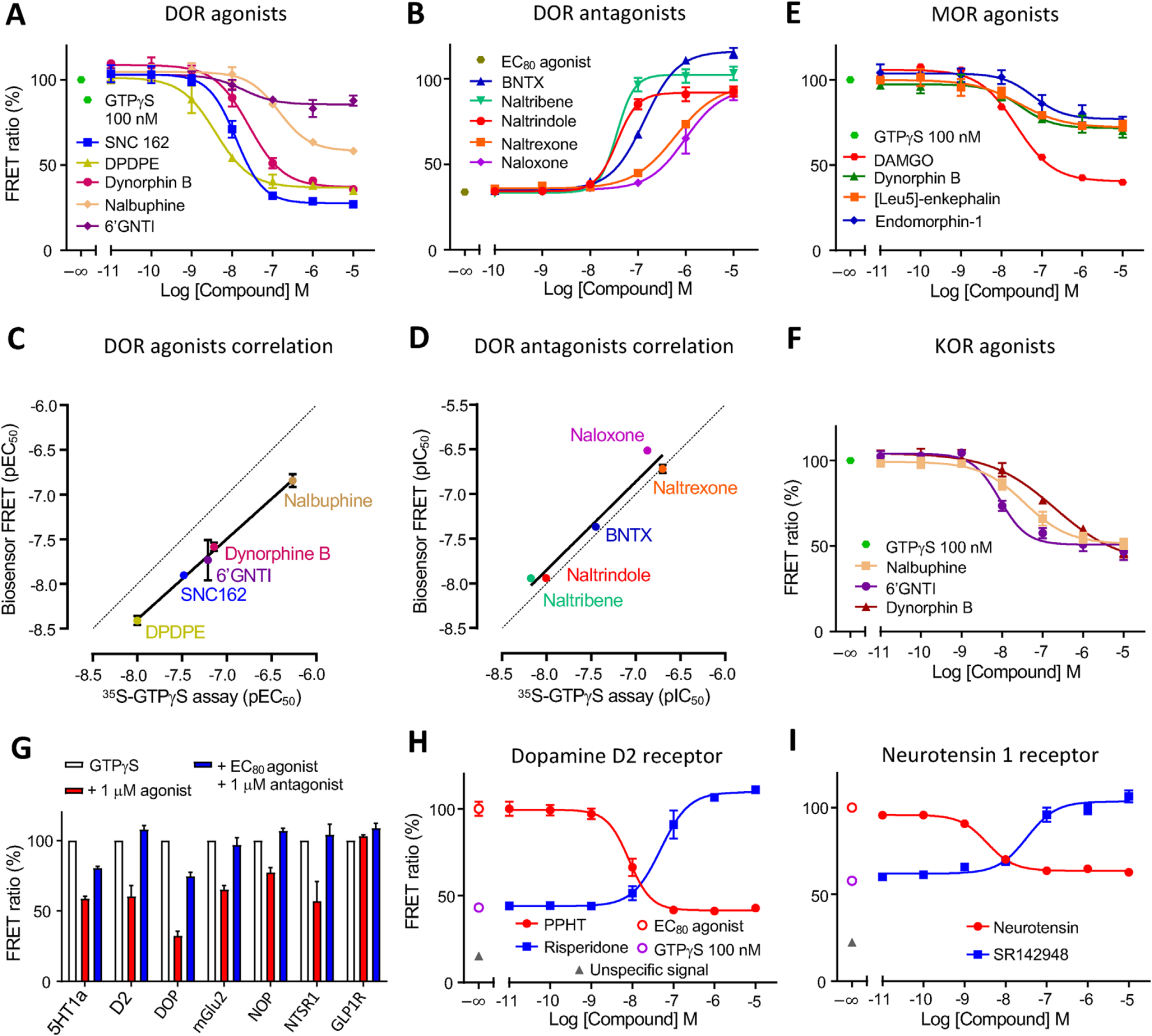
Pharmacology of the opioid receptors using the Gi protein sensor. (A) Measurement of the FRET signal obtained with the sensor peptide‐XL665 + Ab13 for the indicated dose‐response of agonists and partial agonists using the human δ‐opioid receptor (DOR) membrane preparations (10 ng) in presence of 100 nM GTPγS. (B) Similar experiments with the indicated dose‐response of antagonists on DOR membrane preparations (10 ng) in presence of EC_80_ SNC 162 and 100 nM GTPγS. (C,D) Correlation between the Log EC_50_ and Log IC_50_ measured with the FRET sensor peptide‐XL665 + Ab13 for the agonists *(C)* and antagonist *(D)* respectively, and these values obtained from GTPγS assays in literature. (E,F) Similar experiments with the human μ‐opioid receptor (MOR) *(E)* and κ‐opioid receptor (KOR) *(F)* membrane preparations. (G) trFRET signal for the indicated human G_i_‐coupled receptors, and the G_S_‐coupled receptor GLP1R used as a negative control, determined with the sensor peptide‐XL665 + Ab13 in presence of their specific agonists or antagonists, and measured on membrane preparations in presence of 100 nM GTPγS. A significant loss of trFRET signal was observed for all receptors excepted for GLP1R. (H‐I) Measurement of the activation of the endogenous G_i_ proteins by the human dopamine D2 short *(H)* and neurotensin 1 *(I)* receptors in presence of their agonists (red), or antagonists (blue) using the FRET‐based G_i_ protein sensor made of the peptide‐XL665 + Ab13, in membrane preparations (10 ng) in the presence of 100 nM GTPγS. Values are means ± s.e.m. of three independent experiments performed in triplicates.

Interestingly, DOR partial agonists can be discriminated from full agonists (Figure 6A). In addition, antagonists such as BNTX and naltriben, that were described as inverse agonists [48] in contrast to the other antagonists (naltrindole, naltrexone and naloxone) showed a larger efficacy than the other antagonists (Figure 6B). It is consistent with our sensor being able to measure a larger proportion of the G_i_.GTPγS conformation in the presence of inverse agonists, since these ligands are expected to induce less G protein emptying resulting from the constitutive activity of the receptor.

The analysis of the two other opioid receptors, MOR and KOR, reveals that our FRET sensor is also relevant to investigate these receptors (Figure 6E,F). Nalbuphine acts as a full agonist on KOR and a partial agonist on DOR as previously reported [49, 50]. But the dynorphin B reported to be a full agonist on these three opioid receptors, behaves as a partial agonist on µOPr with our sensor [51]. Further studies will be necessary to investigate if these unexpected differences are due to the fact that our sensor recognizes Gα_i1_ and Gα_i3_ only, and not Gα_i2_, or it is due to a biased agonist activity of dynorphin B on the G_i/o_ proteins [52].

Finally, we have applied our FRET sensor to other strictly G_i/o_‐coupled receptors (Figure 6G) such as the nociceptin (**Extended Data** Figure 7), dopamine D2 (Figure 6H), serotonin 5HT_1A_ and mGlu2 receptors. We also investigated the neurotensin receptor (Figure 6I) that has a primary coupling to Gq with a secondary coupling to Gi and Gs proteins [53], and GLP1R that do not activate G_i/o_. For these different receptors, the maximal rate of agonist‐induced Gα_OPEN_ form was estimated between 20% and 40% of the detectable pool of G_i/o_ proteins measured by the tools mAb13 + peptide‐XL665 (Figure 7). As a negative control, no activation has been detected from G_S_‐coupled receptors such as GLP‐1 receptor (Figure 6G). These data nicely illustrate that GPCRs can have different effect on the Gα_OPEN—_GαGTP equilibrium.

**FIGURE 7:**
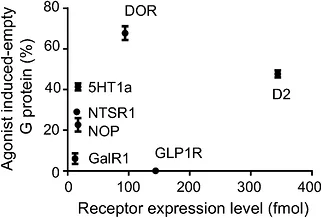
Determination of the amount of agonist‐induced Gα_i_ empty forms for the indicated receptors. Rate of the agonist‐induced Gα_i_ empty determined as in Figure 5 for the indicated receptor (dopamine D2, δ opioid DOR, galanin GalR1, neurotensin NTSR1, nociceptin NOR and serotonin 5HT1a) expressed at different levels in the membrane preparations containing endogenous Gi proteins. Values are means ± s.e.m. of three independent experiments performed in triplicates.

### 2.8 Visualizing G Protein State Equilibria and Ternary Complex Stabilization *in Cellulo*

To confirm that the receptor‐mediated nucleotide release and stabilization of the open state observed in membranes can occur in a more physiological environment, we adapted our trFRET sensors for use in a cellular context using a soft permeabilization approach (Figure 8A). Consistent with our membrane findings, the agonist DAMGO induced a concentration‐dependent decrease in trFRET signal between the GTP bound‐state peptide‐XL665 and the Ab13‐Tb in HEK293 cells expressing µOPr (Figure 8B). This decrease, observed in the presence of both 10 and 100 nM of GTPγS, directly correlates with the receptor‐stimulated release of GTPγS from the Gα subunit, confirming the “reverse” GEF activity in cells.

**FIGURE 8:**
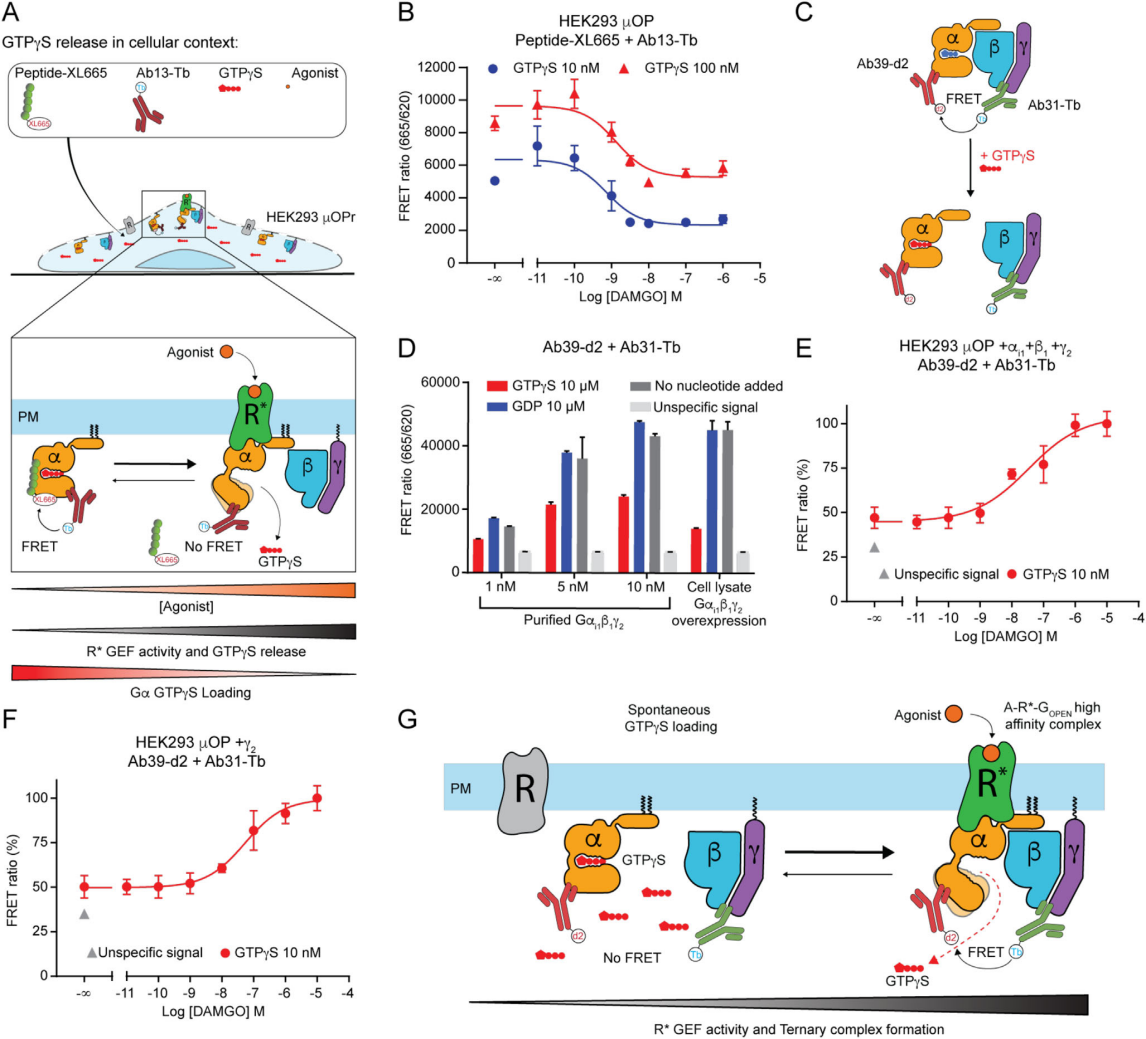
GPCR‐mediated stabilization of the G protein open‐state ternary complex in cells. (A) Schematic of cellular trFRET adaptation via soft permeabilization. (B) DAMGO‐induced GTPγS release in cells measured by the peptide‐XL665/Ab13‐Tb sensor. (C) Design of the heterotrimer sensor (Ab39‐d2/Ab31‐Tb) for tracking Gα/βγ reassociation. (D) Validation of heterotrimer dissociation in purified proteins and lysates. (E‐F) Agonist‐induced increase in trFRET signal in cells, indicating stabilization of the ternary complex at overexpressed (E) and endogenous (F) G protein levels. (G) Proposed model of GPCR‐mediated G protein state equilibria.

To investigate the structural consequences of this release, we developed a novel heterotrimer sensor consisting of Ab39‐d2 targeting Gαi/o and Ab31‐Tb, a new antibody targeting the Gβγ interface (Figure 8C). Characterization of Ab31 revealed that its binding is strictly dependent on the presence of the Gγ2 subunit and shows specificity for Gβ1γ2 and Gβ3γ2 complexes (**Extended Data** Figure 8A–E). Quantitative PCR confirmed that the HEK µOPr cell line lacks endogenous Gγ2 (**Extended Data** Figure 8G–I), necessitating its overexpression to facilitate heterotrimeric G protein complex detection by the sensor.

In purified proteins and cell lysates, the addition of GTPγS induced a robust decrease in trFRET between Gα and Gβγ, reflecting heterotrimer dissociation (Figure 8D). However, in the cellular context, DAMGO stimulation produced a paradoxical increase in trFRET ratio in cells pre‐loaded with 10 nM GTPγS (Figure 8E). This increase indicates that the activated receptor actively recruits dissociated Gβγ back to the Gα subunit by stabilizing the nucleotide‐free open conformation (Gα_OPEN_). We observed a similar phenomenon when only Gγ2 was overexpressed, demonstrating that the sensor can track the stabilization of the high‐affinity ternary complex at endogenous Gαi/o and Gβ expression levels (Figure 8F).

Titration experiments revealed that this observation is critically dependent on the GTPγS concentration; the GEF‐mediated stabilization of the ternary complex is most apparent when GTPγS levels are sufficient to induce basal dissociation but low enough to allow the receptor to shift the equilibrium back to the open state (**Extended Data** Figure 8J,K). Altogether, these data support a model where the activated GPCR acts as a scaffold that not only promotes nucleotide exchange but actively stabilizes a high‐affinity A‐R^*^‐ Gα_OPEN_‐Gβγ complex by promoting the release of bound GTP (Figure 8G).

## 3 Discussion

Our study shows that an active GPCR can not only favor the formation of the GTP bound state, but can also release GTP from the active G protein under certain conditions, to reform a Gα_OPEN_ state offering the possibility to precisely control the amount of GTP‐bound active form generated by the activated GPCR. Such observations were made possible thanks to the innovative tools we report here that allow the detection of either the open or the GTP bound state of endogenous Gi proteins. The validation of these tools is well illustrated by the determination of both the efficacy and potency of various ligands acting at different Gi coupled GPCRs.

Up to now, G protein activation is mainly being measured using over‐expressed biosensors that can possibly interfere with the coupling process [28, 29, 31, 32, 51]. To measure native G protein activation, most assays quantify the GTP bound form of the G protein using either radioactive [26, 27] or fluorescent GTP [33, 34, 35] analogs, or specific sensors responding to GTP bound G proteins [30]. These assays just estimate the quantity of active G proteins. However, the amount of GTP bound form of G proteins is not only controlled by the GTP binding step resulting from GPCR activation, but also by the GTPase activity [54] and its regulation by RGS proteins [7], a step that is not involved when non‐hydrolysable GTP analogs are used. Another process possibly involved in the final amount of GTP‐bound G proteins is the capacity of GPCRs to stabilize the open form of G proteins [4], likely able to release not only bound GDP, but also bound GTP, then possibly limiting the amount of active G proteins. Indeed, such a process has well been reported in the early days [17, 55], but is not yet fully characterized, and its possible influence of GPCR coupling efficacy not yet fully considered [56].

While this manuscript was under preparation, two independent studies from the Bohn's laboratory reported agonist‐dependent GTPγS release at opioid and other Gi/o‐coupled receptors using radiolabeled nucleotide pulse–chase paradigms [19, 20]. These studies provide strong pharmacological evidence that receptor activation can promote dissociation of GTPγS from Gi proteins and further propose that distinct agonists may differentially favor GTP binding vs. GTP release, consistent with multi‐active‐state models of GPCR signaling. Our findings are fully consistent with the existence of receptor‐mediated GTP release, but extend these observations by directly resolving the underlying conformational states of endogenous Gi proteins. By selectively detecting the nucleotide‐free open state and the GTP‐bound active state using independent trFRET sensors, we demonstrate that activated GPCRs actively shift the equilibrium between these states, rather than simply promoting a single unidirectional exchange reaction.

In the present work, we take a complementary approach by directly monitoring the conformational equilibrium of endogenous Gi proteins using trFRET sensors that selectively recognize defined Gαi subunits and discriminate between nucleotide‐free and GTP‐bound conformations. This enables direct assessment of receptor‐induced GTP release from specific G protein subtypes, rather than inference from bulk nucleotide turnover. Such subtype‐level resolution is particularly relevant given that GPCR coupling efficacy, apparent signaling bias, and downstream signaling profiles can depend on the precise Gα isoform engaged. We developed two pairs of binders that allow to selectively detect the open form and the GTP bound form of the native Gαi proteins. Using these tools labelled with trFRET compatible fluorophores, we could measure the effect of the activation of various GPCRs on the formation or disappearance of the three main states of the Gi protein subtypes, the GDP‐bound, the open, and the GTP bound states. As expected, we did detect the increase in open G proteins from pre‐loaded GDP‐bound G proteins.

We also observed the disappearance of the pre‐loaded GTP‐bound G proteins, as well as the increase in the open form, indicating that GPCRs can stabilize the open form of the G proteins, starting from either the GDP‐bound form known to be associated with Gβγ, or the GTP‐bound form, that may not be associated with Gβγ. The large signal to noise ratio of the trFRET changes observed allowed an easy determination of the potencies and efficacies of agonists, as well as the action of selective antagonists. As such, these pharmacological data, fully consistent with the literature, nicely validate our tools to detect native specific Gi protein activation. Because of its high selectivity, this approach can well be expanded to monitor the activation of any specific subtypes of native G proteins. Most importantly, these data also indirectly validate our observation that the active receptors are able to stabilize the open form of the Gi protein from either the GDP‐ or the GTP‐bound forms. This observation is consistent with previous studies revealing the release of GTPγS from the G protein by activated GPCRs [19, 20, 54, 57], and by FRET studies(*50*) showing that strong GPCR activation can lead to the recruitment of the GTP bound G protein on the receptor, and the reformation of the inactive Gαβγ complex in cells.

From a mechanistic standpoint, nucleotide binding and release assays alone cannot distinguish whether observed decreases in GTPγS occupancy arise from altered nucleotide binding kinetics, reassociation of Gα–GTP with the receptor, or stabilization of nucleotide‐free intermediates. By contrast, our conformational sensors reveal that receptor activation consistently increases the population of the open, nucleotide‐free Gi state, irrespective of whether the starting pool of G proteins is GDP‐ or GTP‐loaded. This is consistent with the receptor‐induced GTP release being mechanistically coupled to stabilization of the open G protein conformation. By directly tracking the reassociation of the heterotrimer in cells, we provide structural evidence that the ‘reverse mode’ is not merely a decrease in nucleotide occupancy but a physical stabilization of the high‐affinity A‐R‐Gα_OPEN_/Gβγ ternary complex. Using our heterotrimer sensor, we observed a paradoxical increase in trFRET signal upon agonist stimulation in cells pre‐loaded with GTPγS, indicating that the activated receptor actively recruits dissociated Gβγ back to the Gα subunit. This stabilization of the ternary complex was found to be critically dependent on the nucleotide concentration, becoming most apparent when GTPγS levels permit basal dissociation but allow the receptor to shift the equilibrium back toward the open state. Notably, DAMGO displayed an approximately tenfold higher apparent potency in permeabilized cells than in isolated membrane preparations, highlighting the context dependence of operational receptor–G‐protein coupling. Differences in membrane organization, receptor/G‐protein stoichiometry, endogenous interacting proteins, nucleotide exposure, permeabilization, and sensor accessibility may contribute to this shift. This mechanism likely serves as a fundamental tuning knob for signaling output, where the receptor can quantitatively limit the pool of active, dissociated G proteins under conditions of high‐efficacy activation or restricted GTP availability.

Our observations then demonstrate that the amount of the GTP‐bound active Gi proteins is controlled by three reactions: i) GTP binding on the high affinity open state of the G protein associated with the active GPCR; ii) the intrinsic GTPase activity potentiated by RGS proteins, leading to the formation of the GDP‐bound form; and iii) the capacity of the active GPCR to bind the GTP‐bound state to re‐form the open state of the G protein, then inactivating the G protein. This finding nicely complements previous results by Hommers et al. showing that strong receptor activation can limit the amount of active Gi proteins [56]. Such findings are consistent with the observation that strong activation of the adenosine receptor did not lead to a full activation of GIRK channels, a phenomenon that can be observed in native atrial cells, and indeed led to a paradoxical decrease in pre‐activated GIRK channels. Our findings bring further evidence that the re‐formation of the RG complex from an activated GPCR and the GTP active G protein can occur under certain conditions, leading to a decrease in the maximal amount of active G proteins, then limiting the maximal cellular response. Although it is accepted that the high GTP concentration in the cell (from 50 to 1000 µM) [12] as well as the large amount of G proteins relative to a GPCR would largely favor the formation of the GTP bound state of the G protein, a strong and sustained GPCR activation, especially in a limited subcellular compartment, may be sufficient to decrease GTP concentration to a level allowing the formation of a significant amount of RG complex. This could then limit the maximal signal as observed with the GIRK channel activation [56]. However, we can speculate that this backward reaction may well depend on the receptor and the G protein subtype considered, and possibly on the ligand considered [58], as one can easily expect it will depend on the agonist‐bound receptor affinity for the open state of the G protein. The possibility that certain agonists may bias signaling toward GTP release has been proposed to confer therapeutic advantages in vivo, particularly in the context of opioid analgesia [20]. While these observations are intriguing, they should be interpreted in light of the strong dependence of nucleotide exchange equilibria on receptor density, G protein subtype availability, and intracellular GTP concentrations, parameters that can vary substantially across cellular contexts and experimental systems.

Our results indicate that receptor‐driven stabilization of the open G protein state is an intrinsic property of GPCR–Gi coupling and may therefore operate broadly across receptors, ligands, and G protein subtypes. Rather than representing a rare or ligand‐restricted signaling mode, GTP release appears to be a fundamental component of receptor control over G protein state equilibria, capable of quantitatively limiting signaling output under peculiar conditions of sustained or high‐efficacy receptor activation and limited amount of GTP. Although more work is needed to better understand such a process, our data obtained with native G proteins, and with two independent sets of tools, and consistent with previous studies [19, 20, 56], indicate that it can occur and may limit the maximal response of a GPCR under peculiar conditions that remain to be identified. In support of a likely physiological involvement of such a process, drugs with differential effect on this GPCR action display specific responses in animals [19, 20]. Whatever the possible physiological relevance of this process, our data and tools highlight its usefulness in characterizing the pharmacological properties of GPCRs.

## 4 Methods

### 4.1 Materials

Peptide KB 1753 (SSRGYYHGIWVGEEGRLSR) [43] were synthesized by PolyPeptide group. Fluorophores Lumi4‐Tb and d2 were provided by Revvity (#62TBSPEA, #62D2DPEA). Biotinylation and cysteine addition were performed by Revvity. Commercial antibody against Gα_i_ proteins (SC‐13533) were from Santa Cruz Biotechnology Inc. Home‐made antibodies were provided by Revvity from mice immunization program. Membrane preparations were purchased from Revvity (Membrane Target Systems #RBHODM400UA, #6110549400UA, #6110558400UA, #RBHS1AM400UA, #RBHD2CM400UA, #RBXNT1M400UA, #6110537400UA, #RBHORLM400UA) and Eurofins Pharma Discovery Services. BNTX, DAMGO, DPDPE, dynorphin B, endomorphin‐1, J113397, [Leu^5^]‐enkephalin, naloxone, naltrexone, naltrindole, neurotensin, nociceptin, risperidone, SNC162 and SR142849 were from Tocris Bioscience. Nalbuphine, naltriben, PPHT and 6’GNTI were from Sigma–Aldrich.

### 4.2 Production and Purification of the Recombinant Gα_i1_

The synthetic gene coding for the full‐length human Gα_i1_with a Twin Strep‐Tag (TST) in N‐terminus (SAWSHPQFEKGGGSGGGSGGSAWSHPQFEK) was cloned into baculovirus expression system (Bac‐to‐Bac Baculovirus expression system, Thermo Fischer Scientific). Sf9 insect cells were transfected with bacmid coding for TST‐Gα_i1_ with Cellfectin II reagent and cells were cultured in suspension and incubated at 28°C for 72 h. The supernatant of infected cells was collected as first baculovirus production batch (P1). Then Sf9 cells were infected with the baculovirus P1 and incubated at 28°C for 6 days in order to produce the second baculovirus batch (P2), that have been collected from the supernatant of the cell culture.

Sf9 cells were infected with baculovirus P2 at a multiplicity of infection of 5, at a cell density of 2 × 10^6^ cells/mL and incubated at 28°C for 48 h. Cells were harvested by centrifugation at 4000 rpm for 10 min. All purification steps were carried out at 4°C. Pellets were resuspended with ice‐cold lysis buffer (LB) consisting of 20 mM HEPES (pH 8), 150 mM NaCl, 1 mM DTT, 1 mM EDTA, 23 µg/mL PMSF, 21 µg/mL TLCK, 21 µg/mL TPCK (protease inhibitors), 3.3 µg/mL leupeptin, 3.3 µg/mL Lima bean trypsin inhibitor. Cells were homogenized with Ultra Turrax three times for 15 sec, detergent Genapol 0.05% was added to solubilize membranes, lysed cells were incubated 30 min at 4°C under agitation. Proteins were collected in the supernatant by centrifugation at 38 420 x g at 4°C for 15 min. The clear supernatant was applied on a column containing Strep‐Tactin Superflow resin (IBA) which had been equilibrated with LB. The column was washed with wash buffer (20 mM HEPES pH 8, 150 mM NaCl, 1 mM DTT, 1 mM EDTA, 11 mM CHAPS), and protein were eluted with elution buffer (20 mM HEPES pH 8, 150 mM NaCl, 1 mM DTT, 1 mM EDTA, 11 mM CHAPS, 5 mM D‐desthiobiotin). The fractions containing proteins were pooled and the buffer solution was exchanged with 20 mM HEPES pH 8, 100 mM NaCl, 11 mM CHAPS with Sephadex G25 PD‐10 desalting columns (GE Healthcare Life Sciences). TST‐Gα_i1_ proteins were stored at −80°C.

### 4.3 Bodipy‐GTPγS Binding Assay

Recombinant TST‐Gα_i1_ proteins were tested in Bodipy‐GTPγS (Bodipy‐FL‐GTPγS, Thermo Fischer Scientific) binding assay. Proteins were diluted in HEM buffer (10 mM HEPES, 1 mM EDTA, 10 mM MgCl_2_) and added (10 µL) in a 384‐well black plate (Greiner) at several range of concentrations. Bodipy‐GTPγS at 10 nM was added (5 µL), and kinetic binding of the fluorescent GTPγS was measured at PHERAstar (BMG Labtech) with the following parameters: excitation at 482 nm (10 flashes per well), emission at 520 nm and gain of 300. Nonspecific signal is obtained by adding non labeled GTPγS.

### 4.4 Antibodies and Peptides Binding Assay

The binding assay was performed in 384‐well white plate (Greiner). Recombinant TST‐Gα_i1_ proteins at 1 nM (5 µL) were incubated with 10 µM of each nucleotide (5 µL), GDP or GTPγS within 30 min at 25°C. Then antibodies or peptide labeled with d2 against proteins were added at different concentrations (5 µL). The antibody against the tag of the protein (anti‐TST), labeled with Lumi4‐Tb was added at 0.5 nM (5 µL). Reagents were incubated at 25°C for 3 h. trFRET measurements were measured with a PHERAstar microplate reader (BMG Labtech) with the following setup: excitation at 337 nm (40 flashes per well), donor emission at 620 nm, acceptor emission at 665 nm, integration delay of 60 µs and integration time of 400 µs.

### 4.5 Cell Culture and Transfection

Plasmids encoding for the different G‐proteins with a TST and a Flag‐tag added at the N‐terminus of the proteins (Gα_i1_, Gα_i2_, Gα_i3_, Gα_o_, Gα_z_, Gα_s_, Gα_q_, Gα_12_ and Gα_13_) were synthetized by Genecust. HEK293 cells were cultured in DMEM supplemented with 10% FBS and transfected using Lipofectamine 2000 in 96‐well black plates (Greiner). For each well, the mix ratio DNA (µg): Lipofectamine 2000 (µL) was 0.4. A total of 0.15 µg of DNA was incubated with 0.375 µL of Lipofectamine 2000 in 50 µL Opti‐MEM (1x) (Invitrogen) for 20 min at 25°C and then added in the well. 50 000 cells were added in each well on the mix and incubated at 37°C in a humified incubator containing 5% CO_2_.

### 4.6 Antibodies Selectivity Assay

The HEK293 cells were pre‐incubated for 30 min at 20°C in the presence or absence of nucleotides GDP or GTPγS (10 µM) diluted in permeabilized buffer containing 50 mM Tris HCl pH 7.4, 10 mM NaCl, 10 mM MgCl_2_, 0.1% BSA and 0.02% Triton. Antibodies against the two tags were added to measure the expression levels of each recombinant G proteins (0.5 nM anti‐Twin‐strep‐tag labeled with Lumi4‐Tb (provided by Revvity), 10 nM HTRF Anti‐FLAG mAb d2‐Conjugate (Revvity, #61FGBDLF/A/B). The binding of each antibody anti‐Gα labeled with d2 (10 nM) was measured by the trFRET signal with the anti‐TST labeled with Lumi4‐Tb (0.5 nM) after 20 h incubation at 20°C. A PHERAstar microplate reader (BMG Labtech) was used with the following setup: excitation at 337 nm (40 flashes per well), donor emission at 620 nm, acceptor emission at 665 nm, integration delay of 60 µs and integration time of 400 µs.

### 4.7 [^35^S]‐GTPγS Binding Assay

[^35^S]‐GTPγS binding to membrane preparations were performed in 96‐well microplates (from Revvity). Membrane preparations (15 µg/well) were incubated with or without ligands dose response for 15 min at 30°C in 20 mM HEPES pH 7.4, 100 mM NaCl, 3 µM GDP and 3 mM MgCl_2_. Then, 0.1 nM [^35^S]‐GTPγS (Revvity, #NEG030H250UC) was added to the membranes for 30 min at 30°C. The reaction was stopped by centrifugation (800 g for 10 min) at 4°C and removal of supernatants. Microplates were counted for 2 min per well with the MicroBeta TriLux.

### 4.8 trFRET G_i_ Protein Assay

The activation assay of δ opioid receptor (DOR, 9 pmol/mg), μ opioid receptor (MOR, 8 pmol/mg), κ opioid receptor (KOR, 3.6 pmol/mg), nociceptin receptor (OPRL1, 1.7 pmol/mg), dopamine D2 short receptor (D2sR, 34.4 pmol/mg) and neurotensin receptor (NTS1R, 3.45 pmol/mg) overexpressed in commercial membrane preparations was performed in 384‐well white microplates. All the reagents were diluted in 50 mM Tris HCl pH 7.4, 10 mM NaCl, 10 mM MgCl_2_ and 0.1% BSA. The biotinylated peptide (50 nM biotine‐KB1753DS) was pre‐incubated with the 25 nM HTRF Streptavidin XL665‐Conjugate (Revvity, # 610SAXLF/A/B) for 30 min at 20°C. Then, ligands were diluted with the buffer supplemented with 10 nM GTPγS and incubated with the membrane preparations. At the same time, streptavidin‐peptide complex or d2 labeled antibodies and Lumi4‐Tb labeled antibodies were added to the membrane preparations and ligands for 1 h at 20°C. trFRET signal was measured with a PHERAstar microplate reader (BMG Labtech) with the following setup: excitation at 337 nm (40 flashes per well), donor emission at 620 nm, acceptor emission at 665 nm, integration delay of 60 µs and integration time of 400 µs.

### 4.9 trFRET Cellular Activation and Stabilization Assays

HEK293 cells stably expressing the human µ‐opioid receptor (µOPr) were transfected with Gαi1, Gβ1 and Gγ2 or Gγ2 alone using Lipofectamine 2000. For cellular trFRET, cells were washed with a phosphate‐free buffer and subjected to **soft permeabilization** using Revvity HTRF Permeabilization buffer#1 within the HTRF Cellular GTP/Gi protein binding kit (Revvity, #62CGTPPEG/C). For the heterotrimer stabilization assay, cells were pre‐incubated with indicated concentrations of GTPγS (10 nM) for 30 min to allow for spontaneous G protein dissociation. trFRET binders (Ab39‐d2 and Ab31‐Tb) were added simultaneously with dose‐responses of DAMGO. Plates were incubated for 1 h at 20°C before measurement of trFRET signal with a PHERAstar microplate reader (BMG Labtech) using the following setup: excitation at 337 nm (40 flashes per well), donor emission at 620 nm, acceptor emission at 665 nm, integration delay of 60 µs and integration time of 400 µs.

### 4.10 Quantitative Real‐Time PCR (qPCR)

To characterize the endogenous landscape of G protein subunits, total RNA was extracted from HEK µOPr cells using the RNeasy Mini Kit (Qiagen). cDNA was synthesized and subsequent qPCR was performed using TaqMan technology (Thermo Fisher Scientific) according to the manufacturer's protocols. Specific TaqMan probes were utilized for the relative quantification of all human Gα, Gβ, and Gγ subtypes. The GAPDH gene was employed as the internal housekeeping gene for normalization. Data were analyzed using the 2^−ΔΔCt^ method to determine the percentage expression relative to GAPDH.

## Author Contributions

A.B., M.D., J.S., L.F., L.L., T.R., E.D, E.T., P.R. and J.‐P.P. designed experiments; M.D., J.L. and L.F. performed protein purification, cellular and in vitro assays; L.L., performed peptide synthesis; S.G. performed protein purification; T.D. designed experiments for the calculation of G protein emptying; A.B., M.D, P.R. and J.‐P.P. wrote the manuscript.

## Conflicts of Interest

P.R. and J.‐P.P. are involved in a collaborative team between the CNRS (IGF, Montpellier) and Cisbio Bioassays, an affiliated company with Revvity Health Sciences Inc.

## Supporting information




**Supporting File**: advs77969‐sup‐0001‐SuppMat.docx.

## Data Availability

Materials, protocols and data are available on request.

## References

[1] A. S. Hauser , M. M. Attwood , M. Rask‐Andersen , H. B. Schioth , and D. E. Gloriam , “Trends in GPCR Drug Discovery: New Agents, Targets and Indications,” Nature Reviews Drug Discovery 16, no. 12 (2017): 829–842, 10.1038/nrd.2017.178. PMC6882681 29075003

[2] J. S. Smith , R. J. Lefkowitz , and S. Rajagopal , “Biased Signalling: From Simple Switches to Allosteric Microprocessors,” Nature Reviews Drug Discovery 17, no. 4 (2018): 243–260, 10.1038/nrd.2017.229. PMC5936084 29302067

[3] R. O. Dror , T. J. Mildorf , D. Hilger , et al., “Structural Basis for Nucleotide Exchange in Heterotrimeric G Proteins,” Science 348, no. 6241 (2015): 1361–1365, 10.1126/science.aaa5264. PMC4968074 26089515

[4] D. Hilger , K. K. Kumar , H. Hu , et al., “Structural Insights Into Differences in G Protein Activation by Family A and Family B GPCRs,” Science 369, no. 6503 (2020): aba3373, 10.1126/science.aba3373. PMC7954662 32732395

[5] D. Goricanec , R. Stehle , P. Egloff , et al., “Conformational Dynamics of a G‐Protein α Subunit is Tightly Regulated by Nucleotide Binding,” Proceedings of the National Academy of Sciences 113, no. 26 (2016): E3629, 10.1073/pnas.1604125113. PMC4932968 27298341

[6] Y. Kwon , S. Mehta , M. Clark , et al., “Non‐Canonical β‐Adrenergic Activation of ERK at Endosomes,” Nature 611, no. 7934 (2022): 173–179, 10.1038/s41586-022-05343-3. PMC10031817 36289326

[7] I. Masuho , S. Balaji , B. S. Muntean , et al., “A Global Map of G Protein Signaling Regulation by RGS Proteins,” Cell 183, no. 2 (2020): 503–521.e19, 10.1016/j.cell.2020.08.052. PMC7572916 33007266

[8] S. G. Rasmussen , B. T. DeVree , Y. Zou , et al., “Crystal Structure of the β2 Adrenergic Receptor–Gs Protein Complex,” Nature 477, no. 7366 (2011): 549–555, 10.1038/nature10361. PMC3184188 21772288

[9] X. J. Yao , G. Velez Ruiz , M. R. Whorton , et al., “The Effect of Ligand Efficacy on the Formation and Stability of a GPCR‐G Protein Complex,” Proceedings of the National Academy of Sciences 106, no. 23 (2009): 9501–9506, 10.1073/pnas.0811437106. PMC2685739 19470481

[10] R. Grisshammer , “The Quest for High‐Resolution G Protein‐Coupled Receptor–G Protein Structures,” Proceedings of the National Academy of Sciences 117, no. 13 (2020): 6971–6973, 10.1073/pnas.2002665117. PMC7132303 32179692

[11] A. Koehl , H. Hu , S. Maeda , et al., “Structure of the µ‐Opioid Receptor–Gi Protein Complex,” Nature 558, no. 7711 (2018): 547–552, 10.1038/s41586-018-0219-7. PMC6317904 29899455

[12] T. W. Traut , “Physiological Concentrations of Purines and Pyrimidines,” Molecular and Cellular Biochemistry 140, no. 1 (1994): 1–22, 10.1007/BF00928361. 7877593

[13] N. Bennett and Y. Dupont , “The G‐Protein of Retinal rod Outer Segments (Transducin). Mechanism of Interaction With Rhodopsin and Nucleotides,” Journal of Biological Chemistry 260, no. 7 (1985): 4156–4168, 10.1016/S0021-9258(18)89245-6. 3920215

[14] B. Kohl and K. P. Hofmann , “Temperature Dependence of G‐Protein Activation in photoreceptor membranes. Transient extra metarhodopsin II on Bovine Disk Membranes,” Biophysical Journal 52, no. 2 (1987): 271–277, 10.1016/S0006-3495(87)83214-9. PMC1330078 3117126

[15] R. J. Lefkowitz , “Biochemical Mechanisms of Hormone Receptor‐Effector Coupling. Introduction,” Federation Proceedings 41, no. 10 (1982): 2662. 6286368

[16] P. A. Liebman and E. N. Pugh Jr. , “Gain, speed and sensitivity of GTP binding vs PDE activation in visual excitation,” Vision Research 22, no. 12 (1982): 1475–1480, 10.1016/0042-6989(82)90212-7. 6305023

[17] G. Hilf , C. Kupprion , T. Wieland , and K. H. Jakobs , “Dissociation of Guanosine 5′‐[γ‐thio]Triphosphate From Guanine‐Nucleotide‐Binding Regulatory Proteins in Native Cardiac Membranes,” European Journal of Biochemistry 204, no. 2 (1992): 725–731, 10.1111/j.1432-1033.1992.tb16687.x. 1541285

[18] C. Kupprion , T. Wieland , and K. H. Jakobs , “Receptor‐Stimulated Dissociation of GTP[S] From Gi‐Proteins in Membranes of HL‐60 Cells,” Cellular Signalling 5, no. 4 (1993): 425–433, 10.1016/0898-6568(93)90082-w. 8373724

[19] L. M. Bohn and E. L. Stahl , “Characterization of the GTPγS Release Function of a G Protein‐Coupled Receptor,” Nature Communications 16, no. 1 (2025): 11193, 10.1038/s41467-025-66516-y. PMC12712014 41407695

[20] E. L. Stahl , M. A. Swanson , V. Q. Dang , et al., “GTP Release‐Selective Agonists Prolong Opioid Analgesic Efficacy,” Nature 650, no. 8101 (2026): 500–508, 10.1038/s41586-025-09880-5. PMC12893914 41407861

[21] C. Avet , A. Mancini , B. Breton , et al., “Effector Membrane Translocation Biosensors Reveal G Protein and βarrestin Coupling Profiles of 100 Therapeutically Relevant GPCRs,” Elife 11 (2022): 74101, 10.7554/eLife.74101. PMC9005190 35302493

[22] A. S. Hauser , C. Avet , C. Normand , et al., “Common Coupling Map Advances GPCR‐G Protein Selectivity,” Elife 11 (2022): 74107, 10.7554/eLife.74107. PMC9005189 35302494

[23] A. Inoue , F. Raimondi , F. M. N. Kadji , et al., “Illuminating G‐Protein‐Coupling Selectivity of GPCRs,” Cell 177, no. 7 (2019): 1933–1947.e25, 10.1016/j.cell.2019.04.044. PMC6773469 31160049

[24] N. Okashah , Q. Wan , S. Ghosh , et al., “Variable G Protein Determinants of GPCR Coupling Selectivity,” Proceedings of the National Academy of Sciences 116, no. 24 (2019): 12054–12059, 10.1073/pnas.1905993116. PMC6575158 31142646

[25] P. Kolb , T. Kenakin , S. P. H. Alexander , et al., “Community Guidelines for GPCR Ligand Bias: IUPHAR Review 32,” British Journal of Pharmacology 179, no. 14 (2022): 3651–3674, 10.1111/bph.15811. PMC7612872 35106752

[26] G. Milligan , “Principles: Extending the Utility of [^35^S]GTPγS Binding Assays,” Trends in Pharmacological Sciences 24, no. 2 (2003): 87–90, 10.1016/s0165-6147(02)00027-5. 12559773

[27] P. G. Strange , “Use of the GTPγS ([^35^S]GTPγS and Eu‐GTPγS) Binding Assay for Analysis of Ligand Potency and Efficacy at G Protein‐Coupled Receptors,” British Journal of Pharmacology 161, no. 6 (2010): 1238–1249, 10.1111/j.1476-5381.2010.00963.x. PMC3000650 20662841

[28] M. Bunemann , M. Frank , and M. J. Lohse , “Gi Protein Activation in Intact Cells Involves Subunit Rearrangement Rather Than Dissociation,” Proceedings of the National Academy of Sciences 100, no. 26 (2003): 16077–16082, 10.1073/pnas.2536719100. PMC307695 14673086

[29] C. Gales , J. J. Van Durm , S. Schaak , et al., “Probing the Activation‐Promoted Structural Rearrangements in Preassembled Receptor–G Protein Complexes,” Nature Structural & Molecular Biology 13, no. 9 (2006): 778–786, 10.1038/nsmb1134. 16906158

[30] M. Maziarz , J. C. Park , A. Leyme , et al., “Revealing the Activity of Trimeric G‐proteins in Live Cells With a Versatile Biosensor Design,” Cell 182, no. 3 (2020): 770–785.e16, 10.1016/j.cell.2020.06.020. PMC7415655 32634377

[31] R. H. J. Olsen , J. F. DiBerto , J. G. English , et al., “TRUPATH, An Open‐Source Biosensor Platform for Interrogating the GPCR Transducerome,” Nature Chemical Biology 16, no. 8 (2020): 841–849, 10.1038/s41589-020-0535-8. PMC7648517 32367019

[32] I. Masuho , O. Ostrovskaya , G. M. Kramer , C. D. Jones , K. Xie , and K. A. Martemyanov , “Distinct Profiles of Functional Discrimination Among G Proteins Determine the Actions of G Protein–Coupled Receptors,” Science Signaling 8, no. 405 (2015): ra123, 10.1126/scisignal.aab4068. PMC4886239 26628681

[33] A. Koval , D. Kopein , V. Purvanov , and V. L. Katanaev , “Europium‐Labeled GTP as a General Nonradioactive Substitute for [^35^S]GTPγS in High‐Throughput G Protein Studies,” Analytical Biochemistry 397, no. 2 (2010): 202–207, 10.1016/j.ab.2009.10.028. 19849998

[34] J. Labrecque , V. Anastassov , G. Lau , M. Darkes , R. Mosi , and S. P. Fricker , “The Development of an Europium‐GTP Assay to Quantitate Chemokine Antagonist Interactions for CXCR4 and CCR5,” ASSAY and Drug Development Technologies 3, no. 6 (2005): 637–648, 10.1089/adt.2005.3.637. 16438659

[35] D. P. McEwen , K. R. Gee , H. C. Kang , and R. R. Neubig , “Fluorescent BODIPY‐GTP Analogs: Real‐Time Measurement of Nucleotide Binding to G Proteins,” Analytical Biochemistry 291, no. 1 (2001): 109–117, 10.1006/abio.2001.5011. 11262163

[36] C. Mannoury la Cour , C. Herbelles , V. Pasteau , G. de Nanteuil , and M. J. Millan , “Influence of Positive Allosteric Modulators on GABA B Receptor Coupling in Rat Brain: A Scintillation Proximity Assay Characterisation of G Protein Subtypes,” Journal of Neurochemistry 105, no. 2 (2008): 308–323, 10.1111/j.1471-4159.2007.05131.x. 18021295

[37] C. A. Johnston , F. S. Willard , J. K. Ramer , R. Blaesius , C. N. Roques , and D. P. Siderovski , “State‐Selective Binding Peptides for Heterotrimeric G‐Protein Subunits:Novel Tools for Investigating G‐Protein Signaling Dynamics,” Combinatorial Chemistry & High Throughput Screening 11, no. 5 (2008): 370–381, 10.2174/138620708784534798. PMC2440659 18537558

[38] F. S. Willard and D. P. Siderovski , “The R6A‐1 Peptide Binds to Switch II of Gαi1 but is not a GDP‐Dissociation Inhibitor,” Biochemical and Biophysical Research Communications 339, no. 4 (2006): 1107–1112, 10.1016/j.bbrc.2005.11.132. 16338227

[39] A. L. Schmitz , R. Schrage , E. Gaffal , et al., “A Cell‐Permeable Inhibitor to Trap Gαq Proteins in the Empty Pocket Conformation,” Chemistry & Biology 21, no. 7 (2014): 890–902, 10.1016/j.chembiol.2014.06.003. PMC4337399 25036778

[40] G. H. Westfield , S. G. Rasmussen , M. Su , et al., “Structural Flexibility of the Gαs α‐Helical Domain in the β_2_ ‐Adrenoceptor Gs Complex,” Proceedings of the National Academy of Sciences 108, no. 38 (2011): 16086–16091, 10.1073/pnas.1113645108. PMC3179071 21914848

[41] W. R. Leifert , K. Bailey , T. H. Cooper , A. L. Aloia , R. V. Glatz , and E. J. McMurchie , “Measurement of Heterotrimeric G‐Protein and Regulators of G‐Protein Signaling Interactions by Time‐Resolved Fluorescence Resonance Energy Transfer,” Analytical Biochemistry 355, no. 2 (2006): 201–212, 10.1016/j.ab.2006.04.042. 16729956

[42] J. Meng , C. Xu , P. A. Lafon , et al., “Nanobody‐Based Sensors Reveal a High Proportion of mGlu Heterodimers in the Brain,” Nature Chemical Biology 18, no. 8 (2022): 894–903, 10.1038/s41589-022-01050-2. 35681029

[43] C. A. Johnston , E. S. Lobanova , A. S. Shavkunov , et al., “Minimal Determinants for Binding Activated Gα From the Structure of a Gα i1 −Peptide Dimer,” Biochemistry 45, no. 38 (2006): 11390–11400, 10.1021/bi0613832. PMC2597383 16981699

[44] B. K. Atwood , J. Lopez , J. Wager‐Miller , K. Mackie , and A. Straiker , “Expression of G Protein‐Coupled Receptors and Related Proteins in HEK293, AtT20, BV2, and N18 Cell Lines as Revealed by Microarray Analysis,” BMC Genomics 12, no. 1 (2011): 14, 10.1186/1471-2164-12-14. PMC3024950 21214938

[45] E. Von Moo , K. Harpsoe , A. S. Hauser , et al., “Ligand‐Directed Bias of G Protein Signaling at the Dopamine D2 Receptor,” Cell Chemical Biology 29, no. 2 (2022): 226–238.e4, 10.1016/j.chembiol.2021.07.004. PMC8770702 34302750

[46] N. Audet , C. Gales , E. Archer‐Lahlou , et al., “Bioluminescence Resonance Energy Transfer Assays Reveal Ligand‐Specific Conformational Changes Within Preformed Signaling Complexes Containing δ‐Opioid Receptors and Heterotrimeric G Proteins,” Journal of Biological Chemistry 283, no. 22 (2008): 15078–15088, 10.1074/jbc.M707941200. PMC3258885 18381293

[47] C. Gales , R. V. Rebois , M. Hogue , et al., “Real‐Time Monitoring of Receptor and G‐Protein Interactions in Living Cells,” Nature Methods 2, no. 3 (2005): 177–184, 10.1038/nmeth743. 15782186

[48] C. L. Neilan , H. Akil , J. H. Woods , and J. R. Traynor , “Constitutive Activity of the δ ‐Opioid Receptor Expressed in C6 Glioma Cells: Identification of Non‐Peptide δ ‐Inverse Agonists,” British Journal of Pharmacology 128, no. 3 (1999): 556–562, 10.1038/sj.bjp.0702816. PMC1571659 10516632

[49] M. P. Wentland , R. Lou , Q. Lu , et al., “Syntheses of Novel High Affinity Ligands for Opioid Receptors,” Bioorganic & Medicinal Chemistry Letters 19, no. 8 (2009): 2289–2294, 10.1016/j.bmcl.2009.02.078. PMC2791460 19282177

[50] J. Zhu , L. Y. Luo , J. G. Li , C. Chen , and L. Y. Liu‐Chen , “Activation of the Cloned Human Kappa Opioid Receptor by Agonists Enhances [35S]GTPγS Binding to Membranes: Determination of Potencies and Efficacies of Ligands,” The Journal of Pharmacology and Experimental Therapeutics 282, no. 2 (1997): 676–684, 10.1016/S0022-3565(24)36877-6. 9262330

[51] C. L. Schmid , J. M. Streicher , C. E. Groer , T. A. Munro , L. Zhou , and L. M. Bohn , “Functional Selectivity of 6′‐Guanidinonaltrindole (6′‐GNTI) at κ‐Opioid Receptors in Striatal Neurons,” Journal of Biological Chemistry 288, no. 31 (2013): 22387–22398, 10.1074/jbc.M113.476234. PMC3829329 23775075

[52] L. Zhou , E. L. Stahl , K. M. Lovell , et al., “Characterization of Kappa Opioid Receptor Mediated, Dynorphin‐Stimulated [^35^S]GTPγS Binding in Mouse Striatum for the Evaluation of Selective KOR Ligands in an Endogenous Setting,” Neuropharmacology 99 (2015): 131–141, 10.1016/j.neuropharm.2015.07.001. PMC4655192 26160155

[53] M. Yamada , M. Yamada , M. A. Watson , and E. Richelson , “Neurotensin Stimulates Cyclic AMP Formation in CHO‐rNTR‐10 Cells Expressing the Cloned Rat Neurotensin Receptor,” European Journal of Pharmacology: Molecular Pharmacology 244, no. 1 (1993): 99–101, 10.1016/0922-4106(93)90064-g. 8380559

[54] A. G. Gilman , “G Proteins: Transducers Of Receptor‐Generated Signals,” Annual Review of Biochemistry 56, no. 1 (1987): 615–649, 10.1146/annurev.bi.56.070187.003151. 3113327

[55] T. Michel and R. J. Lefkowitz , “Hormonal Inhibition of Adenylate Cyclase. Alpha 2 Adrenergic Receptors Promote Release of [3H]Guanylylimidodiphosphate From Platelet Membranes,” Journal of Biological Chemistry 257, no. 22 (1982): 13557–13563, 10.1016/S0021-9258(18)33483-5. 6292195

[56] L. G. Hommers , C. Klenk , C. Dees , and M. Bunemann , “G Proteins in Reverse Mode,” Journal of Biological Chemistry 285, no. 11 (2010): 8227–8233, 10.1074/jbc.M109.015388. PMC2832974 20075078

[57] N. A. Lambert , “Dissociation of Heterotrimeric G Proteins in Cells,” Science Signaling 1, no. 25 (2008): re5–re5, 10.1126/scisignal.125re5. 18577758

[58] Y. S. Kim , J. H. Yeon , W. Ko , and B. C. Suh , “Two‐Step Structural Changes in M3 Muscarinic Receptor Activation Rely on the Coupled G_q_ Protein Cycle,” Nature Communications 14, no. 1 (2023): 1276, 10.1038/s41467-023-36911-4. PMC9992711 36882424

