# Supplementary material for: Innovative Approach Reveals How Receptors Control G Protein State Equilibria
Source: Adv Sci (Weinh). 2026 Oct 15:e77969. Online ahead of print. doi: 10.1002/advs.77969 (PMC13670153; doi:10.1002/advs.77969)
Supplement: Supplementary file 1 [file ADVS-9999-e77969-s001.docx]

**Innovative approach reveals how receptors control G protein state equilibria**

Alexandre Bouyssou^1,2,*^, Mélanie Da Silva^1,2,*^, Julien Soulé^1^, Ludovic Fabre^1^, Laurent Lamarque^2^, Rémy Sounier^1^, Thierry Durroux^1^, Sébastien Granier^1^, Thomas Roux^2^, Elodie Dupuis^2^, Eric Trinquet^2#^, Philippe Rondard^1#^ and Jean-Philippe Pin^1#^

^1^ Institut de Génomique Fonctionnelle (IGF), Université de Montpellier, CNRS, Inserm, Montpellier, France.

^2^ Revvity, Codolet, France.

**Supplemental information**

**Supplementary Table 1.** Peptide KB1753 and antibody affinities for the purified TST-tagged Ga_i1_ on the indicated conditions, GTPgS-bound, GDP-bound and open, nucleotide-free conformations.

**Extended Data Figure 1. The pair of antibodies Ab26-Ab39 detects preferentially the open conformation of G_i_ proteins.**

trFRET signal produced by the pair of antibodies Ab26 + Ab39 in presence of the purified G proteins, either Gα_i1_ alone or the heterotrimer Gα_i1_βγ, or the endogenous G proteins of membrane preparations, in the absence or presence of the indicated nucleotides (10 μM) and without or with apyrase (200 mU/mL). Values are means ± s.e.m. of three independent experiments performed in triplicates.

**Extended Data Figure 2. Specificity of the antibodies for the different Gα subtypes.**

(**A**) Principle of the assay to determine the specificity of the antibodies developed against the Gα_i_ proteins. Each TST-tagged Gα subtypes were transfected in HEK293 cells and the trFRET signal was measured between each anti-Gα antibody labeled with acceptor and the anti-TST antibody labeled with donor in cell lysates. (**B-D**) trFRET signal between the antibodies Ab13 *(B)*, Ab39 *(C)* and Ab26 *(D)* labeled with acceptor with the anti-TST antibody labeled with donor according to the assay described in *panel A*. The trFRET signal is normalized by the expression of the recombinant Flag-TST tagged Gα proteins determined in *panel e* below. The specificity of the three antibodies is measured in presence or absence of the indicated nucleotides (10 μM). (**E**) Relative quantification of the different Gα subtypes transfected in HEK293 cells and measured by the trFRET signal between the anti-TST antibody and the anti-Flag antibody thanks to the presence of both the TST and Flag epitopes at the N-terminal part of each Gα subunits, in presence or absence of the indicated nucleotides (10 μM). (**F**) Scheme illustrating the principle of the quantification assay developed to determine the relative expression of the different Gα subtypes.

**Extended Data Figure 3. Antibodies and peptide competition assays for binding on the purified TST tagged Gα_i1_ protein.**

(**A**) Binding of the antibody Ab13 labelled with the acceptor (d2) on the purified TST-tagged Gα_i1_ protein bound to an anti-TST antibody labeled with a donor (Tb) in absence or presence of different concentrations of the unlabeled antibodies Ab39 or Ab26 or the unlabeled peptide KB1753DS and measured by trFRET. The competition with the unlabeled peptide has been done in presence of a saturating concentration of GTPγS. (**B**) Similar experiment for the binding of the antibody Ab39 labelled with the acceptor (d2) measured by the trFRET signal with the anti-TST antibody labeled with donor in absence or presence of different concentrations of the unlabeled antibodies Ab13 or Ab26 or the unlabeled peptide KB1753DS. (**C**) Similar experiment for the binding of the antibody Ab26 labelled with the acceptor (d2) measured by the trFRET signal with the anti-TST antibody labeled with donor in absence or presence of different concentrations of the unlabeled antibodies Ab39. Values are means ± s.e.m. of three independent experiments performed in triplicates.

**Extended Data Figure 4. Epitope mapping**.

(**A**) Alignment of residues non conserved within the Gα_i/o_ subunits and description of the mutants made in the Gα_i1_ subunit. (**B**) Structure of Gα_i1_ with the position of the different residues changed in the mutants has been highlighted. (**C**) Binding of the antibody Ab13 labelled with the acceptor (d2) on the indicated TST-tagged Gα_i1_ protein mutants transfected in HEK293 cells and measured by the trFRET signal with an anti-TST antibody labeled with a donor (Tb) in cell lysates. The trFRET signal is normalized by the expression of the recombinant Flag-TST tagged Gα_i1_ protein mutant. **(D-E)** Similar experiments for the antibodies Ab26 *(D)* and Ab39 *(E)* using the same TST-tagged Gα_i1_ protein mutants transfected in HEK293 cells as described in Extended Data Figure 2 *panel A*. **(F)** Affinity values obtained in the binding in *panel c-e* for the three antibodies on the indicated mutants. NB, no binding.

**Extended Data Figure 5. The G_i_ protein FRET sensor can measure the activation of endogenous Gi proteins from different mammalian species.**

Measurement of the activation of the endogenous G_i_ proteins by the human δ-opioid receptor in presence of the agonist SNC162, or the antagonist naltrindole, using the FRET-based G_i_ protein sensor made of the peptide-XL665 and the Ab13 labeled with a donor, in membrane preparations from three indicated mammalian cell type species, HEK293 (human), CHEM-1 (rat) and CHO (hamster). The experiments were performed in presence of 100 nM GTPγS and 10 ng of membrane preparations. Values are means ± s.e.m. of three independent experiments performed in triplicates.

**Extended Data Figure 6. Effects of the peptide KB1773 and the antibodies on the binding kinetics of Bodipy-GTPγS on the purified TST-Gα_i1_.**

(**A**) Binding kinetics of Bodipy-GTPγS (10 nM) on the purified TST-Gα_i1_ (1 μM) in the presence of the peptide-XL665 (1 μM) or antibody Ab13 (1 μM) used alone or in combination. The dissociation kinetics of the Bodipy-GTPγS start at t = 10 min (arrow) after addition of an excess of unlabeled GTPγS (100 μM). (**B**) Similar binding experiments with the antibodies Ab26 (1 μM) or Ab39 (1 μM) used alone or in combination.

**Extended Data Figure 7. Pharmacology of the nociceptin receptor using the Gi protein sensor.**

Measurement of the FRET signal obtained with the sensor peptide-XL665 + Ab13 for the dose-response of the indicated agonist and antagonist using membrane preparations (10 ng) in presence of 100 nM GTPγS. Values are means ± s.e.m. of three independent experiments performed in triplicates.

### **Extended Data Figure 8. Characterization of the Ab39/Ab31 heterotrimer sensor and endogenous subunit landscape.**

**(A-E**) Ab31 binding validation and Gβ isoform specificity. trFRET signal between donor labeled anti-TST tag antibody on Gβ (A, C) or anti-HA tag on Gγ (B, D) and Ab31-d2 in cell lysates. Binding is strictly dependent on the assembly of the Gβγ complex (A-D). Ab31-d2 selectively recognizes Gβ1γ2 and Gβ3γ2 subtypes (E). **(F)** Heterotrimer sensor performance in cellular contexts. Measurement of trFRET signal between Ab39-d2 and Ab31-Tb in HEK293 µOPr and HEK293 CLS cell lines following overexpression of indicated G protein subunits. Robust heterotrimeric G protein detection is contingent on the availability of the Gγ2 subunit. **(G-I)** Endogenous G protein expression profile. Relative mRNA expression of human Gα (G), Gβ (H), and Gγ (I) subtypes determined via TaqMan™ qPCR and normalized to GAPDH. Results confirm the absence of endogenous Gγ2 in HEK µOPr cells. **(J-K)** Optimization of ternary complex stabilization assays. GTPγS titration on the trFRET heterotrimer sensor with or without 10 µM DAMGO on cells overexpressing G αi1β1γ2 (J) or Gγ2 alone (K). These data defined the 10 nM GTPγS concentration required to observe receptor-mediated stabilization of the ternary complex.
